# Characteristics of measles epidemics in China (1951-2004) and implications for elimination: A case study of three key locations

**DOI:** 10.1371/journal.pcbi.1006806

**Published:** 2019-02-04

**Authors:** Wan Yang, Juan Li, Jeffrey Shaman

**Affiliations:** 1 Department of Epidemiology, Mailman School of Public Health, Columbia University, New York, New York, United States; 2 Beijing Center for Disease Control and Prevention, Beijing, China; 3 Department of Environmental Health Sciences, Mailman School of Public Health, Columbia University, New York, New York, United States; The Pennsylvania State University, UNITED STATES

## Abstract

Measles is a highly infectious, severe viral disease. The disease is targeted for global eradication; however, this result has proven challenging. In China, where countrywide vaccination coverage for the last decade has been above 95% (the threshold for measles elimination), measles continues to cause large epidemics. To diagnose factors contributing to the persistency of measles, here we develop a model-inference system to infer measles transmission dynamics in China. The model-inference system uses demographic and vaccination data for each year as model inputs to directly account for changing population dynamics (including births, deaths, migrations, and vaccination). In addition, it simultaneously estimates unobserved model variables and parameters based on incidence data. When fitted to yearly incidence data for the entire population, it is able to accurately estimate independent, out-of-sample age-specific incidence. Using this validated model-inference system, we are thus able to estimate epidemiological and demographical characteristics key to measles transmission during 1951–2004 for three key locations in China, including its capital Beijing. These characteristics include age-specific population susceptibility and incidence rates, the basic reproductive number (*R*_*0*_), reporting rate, population mixing intensity, and amplitude of seasonality. Key differences among the three sites reveal population and epidemiological characteristics crucial for understanding the current persistence of measles epidemics in China. We also discuss the implications our findings have for future elimination strategies.

## Author summary

Despite high vaccine coverage, measles continues to cause large epidemics in China, a country currently supporting 18% of the world's population. To improve understanding of this phenomenon, here we develop a comprehensive model-inference system; using this system, we are able to simulate measles epidemic dynamics and estimate key epidemiological characteristics in three key locations in China during 1951–2004, a period that spans the pre-vaccine and modern mass-vaccination eras. These estimates include spatiotemporal variations in population susceptibility and the basic reproductive number (*R*_*0*_), an epidemiological parameter commonly used to inform target vaccination levels for measles elimination. Our findings reveal population and epidemiological characteristics crucial for understanding the current persistence of measles epidemics in China and for devising future elimination strategies.

## Introduction

Measles is a highly contagious viral disease. Before the availability of an effective vaccine in the 1960s, it infected nearly all children under 15 yr of age. Due to mass vaccination, the number of measles infections has declined dramatically worldwide and in the Americas, elimination of endemic measles transmission was declared in 2016 [1,2]. Given these encouraging outcomes, measles has been targeted for global eradication. Under the Global Vaccine Action Plan, all six World Health Organization (WHO) regions have committed to eliminate measles and five have aimed to achieve this by 2020 [1]. However, as Year 2020 draws close, eradicating measles has proven challenging.

In particular, in China, where reported countrywide vaccination coverage for the last decade has been above 95%—the critical vaccination rate for measles elimination [1,3]—measles continues to cause large epidemics every year [4]. To identify the key factors contributing to this persistent transmission, we recently analyzed the geospatial pattern of measles transmission in China during 2005–2014 [5]. Our study showed that industrial cities tended to sustain endemic measles transmission and higher incidence rates and that the large migrant populations attracted to these cities may facilitate this persistence. However, the mechanism controlling these epidemiologic dynamics remains unclear and longer-term dynamics—in particular, in the years leading to the recent decade—are needed for further diagnosis.

In China, routine disease surveillance has been in place since the 1950s. During 1951–2004, measles cases were recorded by the National Notifiable Diseases Reporting System (NNDRS) nationwide in monthly intervals [6]. However, published data on measles at best reported incidence in yearly intervals and/or monthly numbers aggregated across multiple years. Further, the dynamics of measles are complexly shaped by population susceptibility (due to newborns, migration, natural infection, or vaccination) and contact patterns (e.g., mixing in schools)[7–17], many of which have undergone dramatic changes in China since the establishment of P.R. China in 1949. In particular, the birthrate has changed from over 3% in the 1950s to ~1% in recent years due to changing population policies [18]. Second, as school systems were gradually built and strengthened, the effect of mixing in schools on measles transmission evolved. Third, measles vaccination was introduced in China in 1965 and since then the level of coverage and vaccination procedures (e.g., 1 versus 2 or more doses) have varied over time [6,19]. Lastly, several major social political changes (in particular, the Great Chinese Famine in 1959–1961 [20], the Cultural Revolution in 1966–1976 [21], and Economic Reform since 1978 [22]) have profoundly affected population dynamics and in turn the epidemic dynamics of measles, as well as the quality of surveillance data. These changes altogether create challenges for studying the long-term dynamics of measles epidemics in China.

In this study, we compiled measles incidence data during 1951–2004, as reported in the literature for three locations—Beijing, Guangzhou, and Shandong—with relatively complete population and vaccination data during the same time period (S1 Table). Both Beijing and Guangzhou are highly developed cities in China, whereas Shandong is a province of median economic development (ranked 18^th^ by per capita GDP among 28 provincial level administrative units in mainland China in 1978). As such, comparison of the long-term dynamics between the two cities (Beijing and Guangzhou) and Shandong (as a “control”) provides further insight into the underlying mechanisms governing recent epidemic dynamics in China. Given the sparsity of data, here we develop a model-inference system to infer measles transmission dynamics. Our model-inference system uses demographic and vaccination data over 1951–2004 as model inputs to directly account for changing population dynamics (including births, deaths, migration, and vaccination); in addition, it estimates the unobserved time-varying epidemiological parameters, including the basic reproductive number (*R*_*0*_), population mixing patterns, local seasonality, and reporting rates, based on incidence data. When fitted to yearly incidence data for the entire population, the model-inference system is able to accurately estimate held-out age-specific data (i.e. out-of-sample data) reported for Beijing and Shandong. As such, we are able to recreate measles transmission dynamics in the three study locations during 1951–2004 in great detail (i.e. weekly age-grouped estimates as opposed to the yearly data for the entire population).

## Results

### Comparison of the long-term measles dynamics and seasonality in the study locations

**Fig 1:**
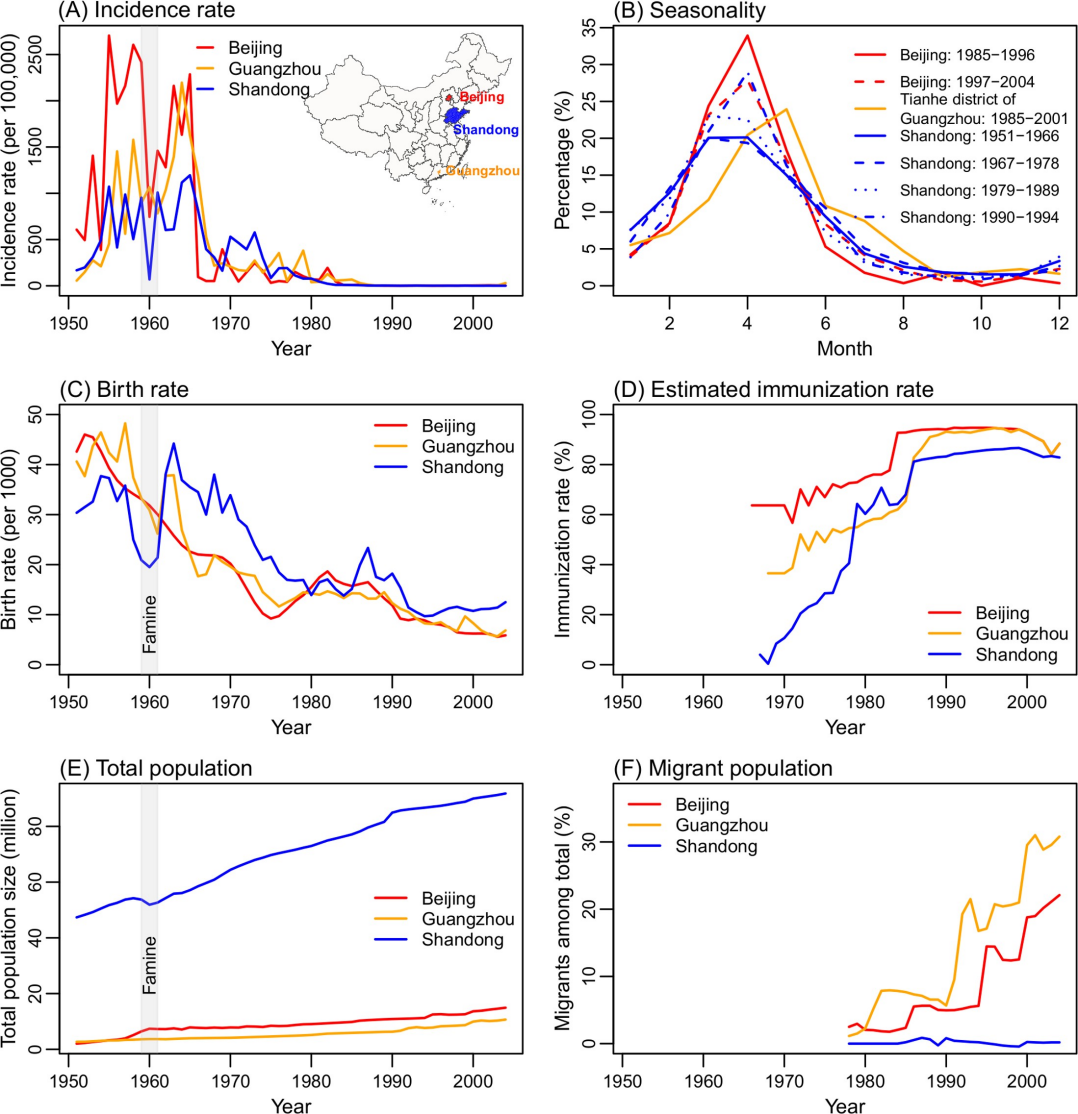
Key measles surveillance data and population variables in the three study locations. (A) Yearly incidence rates during 1951–2004. (B) Seasonal epidemic patterns. (C) Birthrates. (D) Immunization rates, estimated by combining coverage of 2-doses measles vaccine and vaccine efficacy (80% efficacy for 1-dose and 95% for 2-doses; see S1 Text for detail). Note that estimated immunization rates were lower in 2003 and 2004 in Beijing and Guangzhou due to the SARS epidemic. (E) Total population size. (F) The percentage of migrants among total population.

Fig 1 shows the incidence rates in the three study locations—Beijing, Guangzhou, and Shandong—during 1951–2004. During 1951–1965, measles caused large epidemics in all three sites. Unlike the common biennial cycle observed in developed countries [14,23], measles epidemics occurred almost every year, although a transient biennial cycle was evident in Guangzhou and Shandong in the late 1950s (Fig 1A). These frequent epidemics were likely fueled by the high birthrates at the time (Fig 1C). Vaccination programs started in the late 1960s, which dramatically reduced measles incidence; however, the level of vaccine coverage in the early phase (1966–1977) varied greatly among the three sites, with moderate coverage in Beijing but much lower coverage in Guangzhou and Shandong. Following the implementation of nationwide mandatory vaccination (1-dose during 1978–1985 and 2-doses since 1986), the gaps in vaccination started to close. Accounting for effectiveness and dose of vaccination, we estimate that immunization rates (i.e. effective vaccination) surpassed 80% by 1986 and have increased steadily since (Fig 1D). This increasing immunization rate clearly contributed to the continuous decline in measles incidence from 1978 to the early 2000s.

Limited monthly incidence data aggregated over approximately a decade (referred to as "quasi-decadal monthly incidence" hereafter) indicate that, during our study period (1951–2004), measles epidemics tended to start in Nov/Dec, peak in March/April, and last until July in all three locations (Fig 1B). However, epidemics peaked earliest and highest in Beijing, which is located northernmost among the three sites, followed by Shandong, and then ~1–2 months later in Guangzhou, which is southernmost (Fig 1A inset). In addition, measles in Shandong shows a slight shift to later in the year over time (i.e., from peaking in March during 1951–1989 to April during 1990–1994) as epidemic intensity declined due to mass vaccination.

### Model-inference system and validation

**Fig 2:**
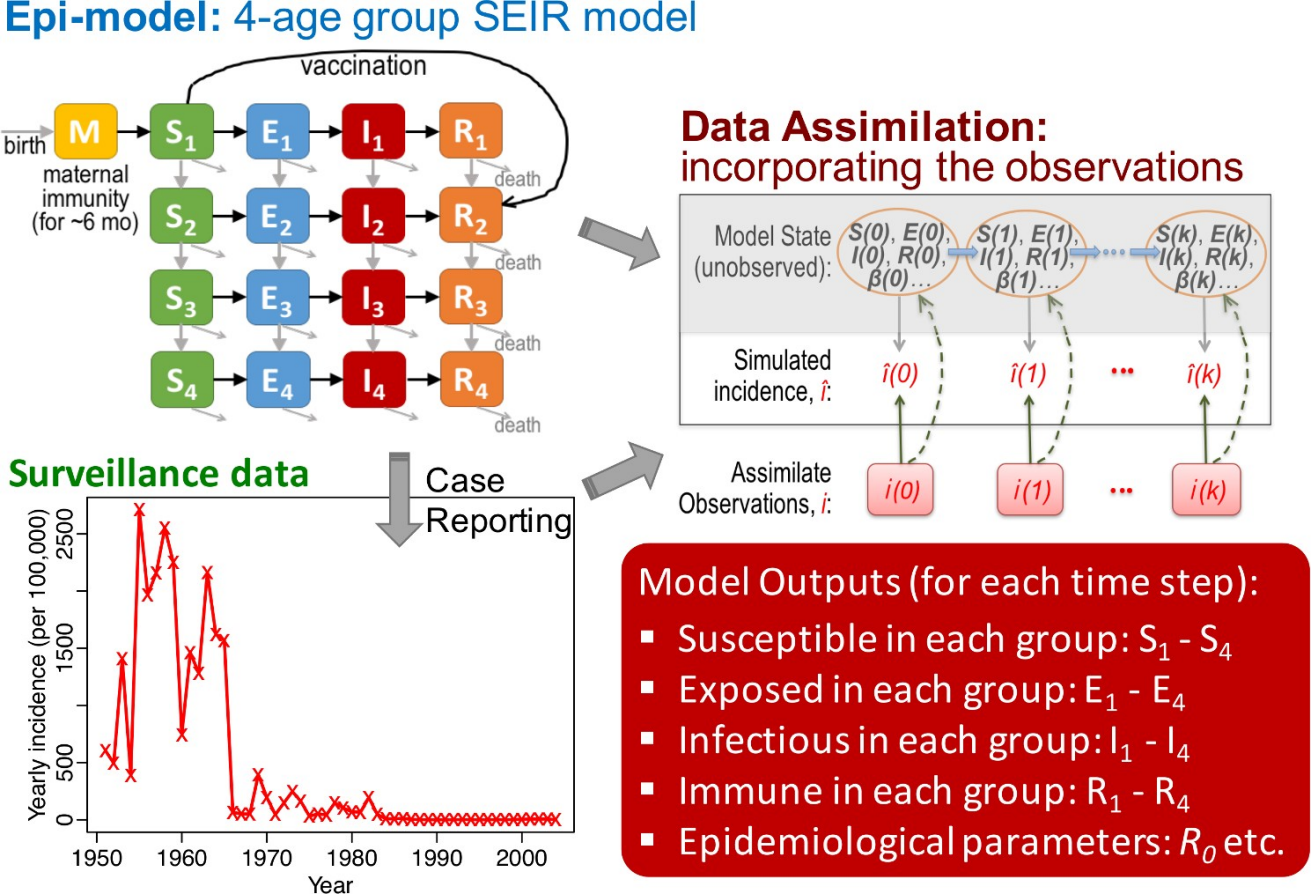
Model-inference system. Our model-inference system is comprised of three components: 1) a model describing the transmission dynamics (here a 4-age group susceptible-exposed-infectious-recovered, SEIR, model); 2) surveillance data (yearly incidence data); and 3) a data assimilation method that recursively incorporates the observations to calibrate the model system; this process directly adjusts the observed variable (solid upward green arrows) and indirectly adjusts unobserved variables/parameters (dashed upward green arrows) in the model.

To infer the transmission dynamics of measles over the five decades, we developed a model-inference system (Fig 2). Our epidemic model uses demographic data (birthrates, age-specific death rates, and migrations) and vaccination data (including vaccine coverage, doses and efficacy) as inputs to account for the aforementioned societal changes during 1951–2004. However, some state variables (e.g. population susceptibility) and parameters (e.g. reporting rate and population mixing pattern) are not observed or documented. To estimate these variables/parameters and changes over time, the model is run in conjunction with a modified particle filter [24,25], a Bayesian inference method. This combined model-inference system simultaneously estimates all state variables and parameters based on yearly incidence for the entire population, the most complete measles data for all three study sites.

We first tested the model-inference system using model-generated mock epidemics (i.e. synthetic testing). When selected by the fits to quasi-decadal monthly incidence, the model-inference system was not only able to reproduce the yearly incidence curve for the entire population (used as observations in the filter) and monthly quasi-decadal incidence (used to select the priors), but also the detailed *weekly* epidemic curves for both the entire population and the key age group (i.e. 1–14 yr olds; S3–S6 Figs). The correlation between the latter two model-simulated time series (2817 weeks over 54 years) and the truths (not used in the filter or selection) was >0.89 for the entire population and >0.87 for the 1–14 yr olds for all tests (S2 Table). In addition, the model-inference system was able to identify the optimal prior state-space that spans the true parameter values (e.g., S3I–S3R Fig for truth 1), in particular, the amplitudes of school forcing and seasonality. For the basic reproductive number (*R*_*0*_), reporting rate, and the mixing parameter *m*_*2*_ (i.e., the exponent of the infectious; see Eq 1 in Methods), collinearity among the three parameters could lead to compensation of one for another (e.g. higher *R*_*0*_ with lower *m*_*2*_, S3I and S3J Fig); however, in general the posterior 95% credible intervals (95% CIs) capture the true values. Further, this issue was mostly seen in two of the tests (i.e., truths 1 and 3) and less severe for the other two tests (S3–S6 Figs). Taken together, these results indicate that our model-inference system is able to truthfully infer the underlying dynamics and key epidemiological parameters, using only the yearly incidence data for filtering and quasi-decadal monthly incidence for selection of parameter priors.

### Model fits of measles transmission dynamics in Beijing, Guangzhou, and Shandong

**Fig 3:**
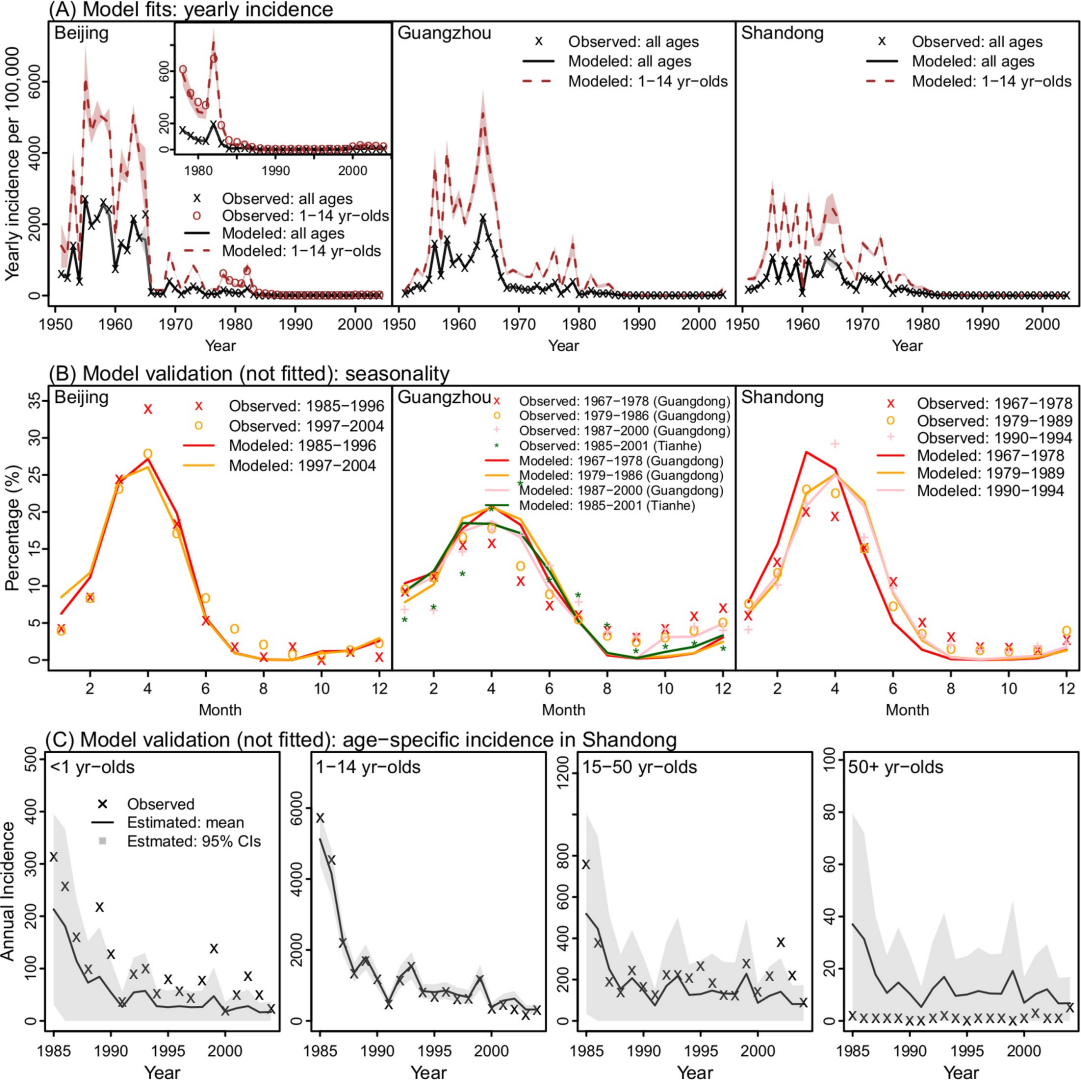
Model fittings of observations in Beijing, Guangzhou, and Shandong. (A) Model estimates of yearly incidence for the entire population and children 1–14 yr of age, compared to observations. Shaded areas (overall incidence in grey and incidence among children in brown) show the 95% Credible Intervals (CIs); some years are not clearly visible due to the narrow range. For Beijing, incidence data for children (1978–2004) are also shown for comparison with model estimates; inset shows model fits for more recent years when incidence was much lower. (B) Model estimated seasonal patterns as compared to available quasi-decadal monthly incidence. Note these data were used for selection of parameter priors but not used directly for model fitting (see Methods). (C) Model estimated age-specific yearly incidence as compared to observations in Shandong. The observations are numbers of measles cases in different ages reported in S. Li et al. 2017 and were *not* used in model fitting or optimization (i.e. out-of-sample data).

We then used the model-inference system to infer the transmission dynamics of measles during 1951–2004 for each site. Fitted to yearly incidence for the entire population only, the model-inference system is able to recreate the observed epidemic curves for Beijing, Guangzhou, and Shandong (Fig 3A) as well as capture the seasonal dynamics as indicated by the quasi-decadal monthly incidence (Fig 3B). More importantly, the model-inference system is also able to accurately predict independent, out-of-sample incidence data reported for different age groups. Compared to data reported for Shandong [26], the correlations (*r*) between the model-estimates and observations during 1985–2004 are, respectively, 0.94, 1.00, and 0.82 for <1, 1–14, and 15–50 yr olds, the three most affected age groups (Fig 3C). Similarly, it accurately predicts out-of-sample yearly incidence for children 1–14 yrs in Beijing (*r* = 0.99; Fig 3A, 1^st^ panel, inset). However, we note that estimates for infants (<1 yr) tended to be slightly lower than reported (Fig 3C, 1^st^ panel). This is expected, as, for simplicity, our model assumed the same reporting rate for all age-groups whereas the reporting rate for infants was likely higher than average. These accurate out-of-sample predictions suggest that the model-inference system is able to correctly capture intra-year transmission dynamics, infection age-structure, and observation errors.

### Inference of measles transmission dynamics in Beijing, Guangzhou, and Shandong

**Fig 4:**
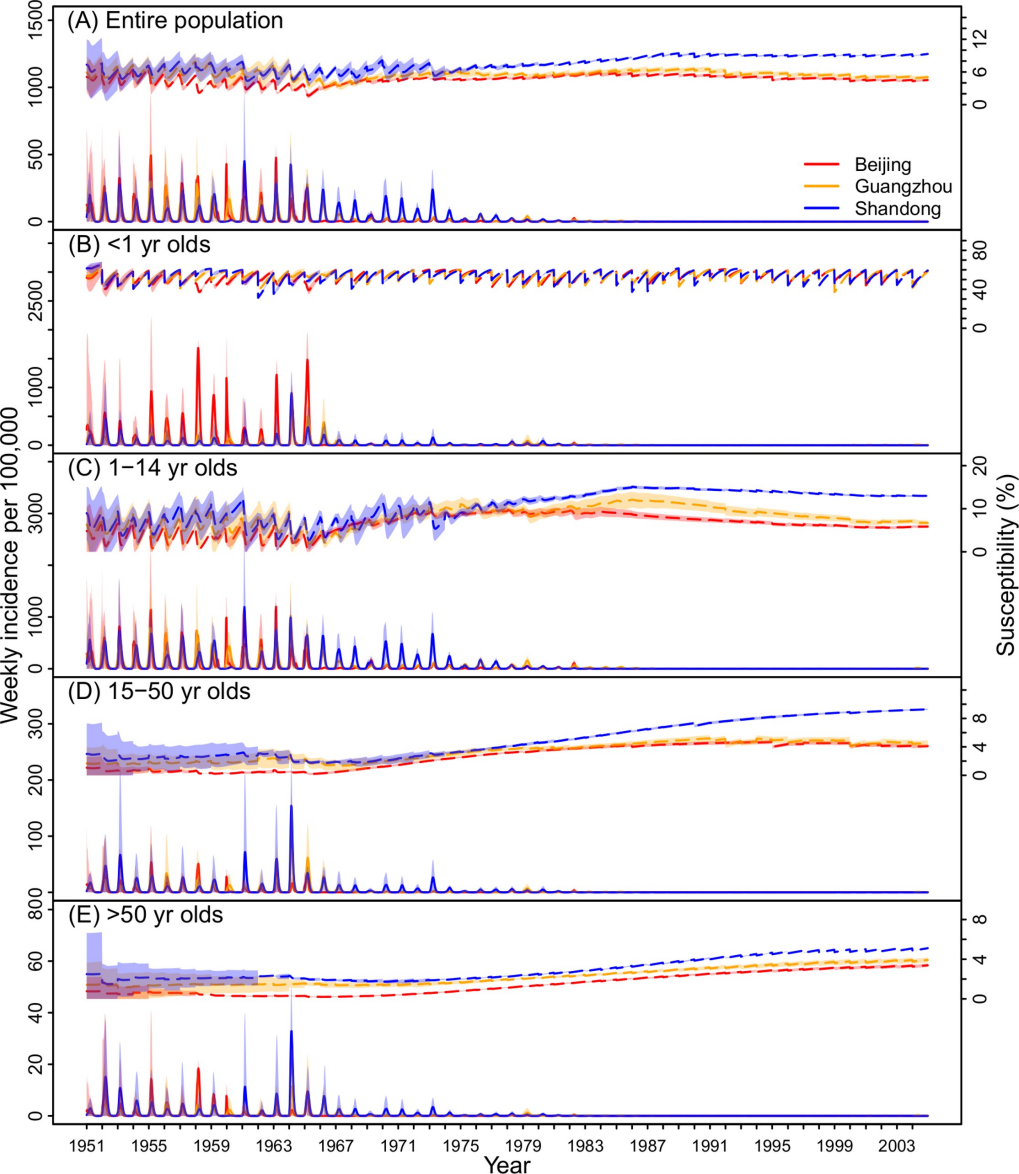
Estimates of key model variables during 1951–2004. Estimated weekly total incidence (solid, y-axis on the left) and population susceptibility (dashed, y-axis on the right) in Beijing, Guangzhou, and Shandong for (A) the entire population, (B) <1 yr olds, (C) 1–14 yr olds, (D) 15–50 yr olds, and (E) >50 yr olds. Shaded areas show the 95% CIs.

With this validated model-inference system, we are thus able to provide detailed estimates of underlying measles transmission dynamics. While the reported incidence rates in Beijing were about twice as high as in Guangzhou and Shandong during 1951–1965 (Fig 1A), after accounting for reporting rate, the total incidence rates (i.e. including unreported cases) were comparable among the three locations during that period (Fig 4A): 2811 (range: 1619–4116) in Beijing, 2978 (2098–4218) in Guangzhou, and 2653 (260–4222) in Shandong per 100,000 population per year. After the introduction of vaccine in the late 1960s, incidence in the two cities declined precipitously. In comparison, due to lower vaccination coverage, in Shandong measles continued to cause large epidemics until the late 1970s.

In addition to inferring total incidence, our model-inference system is also able to estimate population susceptibility during the five-decade record (Fig 4). Before 1966, large epidemics led to similar low susceptibilities in all three locations. The model-inference system estimates that average susceptibilities were 3.9% (2.3–5.1%) in Beijing, 5.8% (4.2–6.7%) in Guangzhou, and 6.1% (5.1–7.0%) in Shandong during 1951–1965. With mass vaccination, susceptibility is determined by both natural infection and immunization. Thanks to high vaccination coverage (Fig 1D), population susceptibility in Beijing and Guangzhou remained at similar low levels despite much lower epidemic intensity (Fig 4A). In comparison, due to lower vaccination coverage and fewer infections, population susceptibility in Shandong increased substantially. The model-inference system estimates that during 1995–2004, population susceptibility in Shandong increased to 9.0% (9.0–9.1%), twice as high as in the other two locations. This large difference in susceptibility was estimated for all ages >1 yr (Fig 4C–4E), in particular for children (13.3%, 13.0–13.9%, Fig 4C) and young adults (8.8%, 8.3–9.2%, Fig 4D).

### Comparison of key epidemiological parameters

**Fig 5:**
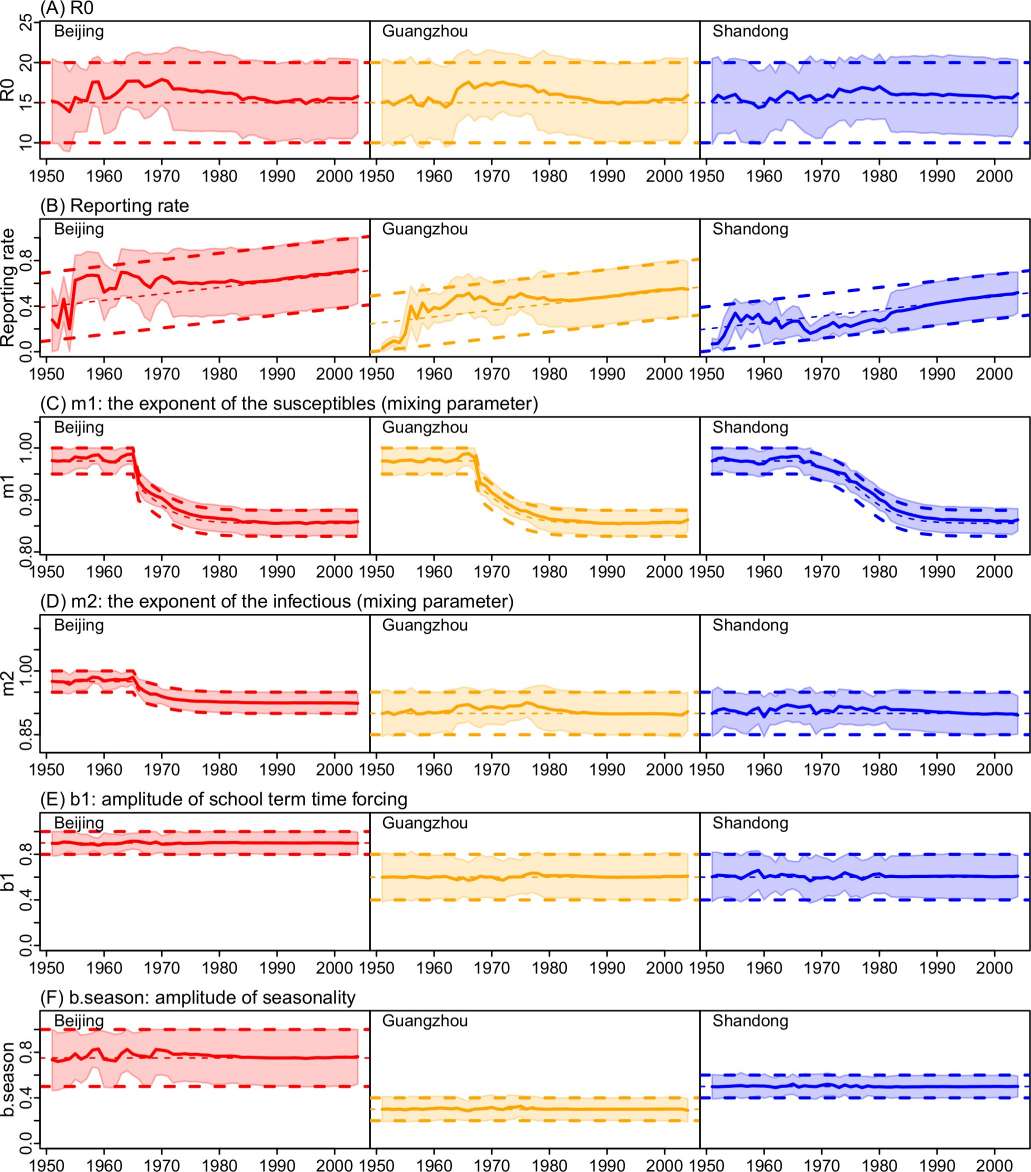
Estimates of key model parameters during 1951–2004. (A) *R*_*0*_; (B) Reporting rate; (C) Mixing parameter *m*_*1*_; (D) Mixing parameter *m*_*2*_; (E) Amplitude of school term time forcing (*b*_*1*_); and (F) Amplitude of seasonality (*b*.*season*). Solid lines show the mean posterior estimates and shaded areas show the 95% CIs; thick dashed lines show the prior ranges and thin dashed lines show the mean values of the priors. Estimates for other parameters are shown in S1 Fig.

Fig 5 shows the estimated spatial temporal variations in key epidemiological parameters over the five decades. Key epidemiological parameters describe the underlying transmission characteristics of an infection. For example, the basic reproductive number (*R*_*0*_), defined as the average number of secondary infections caused by a primary case in a naïve population, indicates the transmissibility of an infection. For measles, *R*_*0*_ estimates are in the range of 12–18 [3], mostly based on epidemics in industrialized countries prior to mass-vaccination. Here we estimate that the mean of *R*_*0*_ was near 16 for most of the years during 1951–2004 and stayed at similar levels after the implementation of mass-vaccination in all three sites in China (see Fig 5A for the full range of *R*_*0*_ estimates). The *mean estimates* over our study period are 16.0 ± 0.9 (Mean ± SD) in Beijing, 15.8 ± 0.9 in Guangzhou, and 15.9 ± 0.5 in Shandong, respectively. These estimates account for under-reporting. Estimated reporting rates, though highest in Beijing and lowest in Shandong, have increased substantially over the five decades, reaching 72% [42%, 100%] (Mean and 95%CI), 55% [30%, 79%], and 52% [34%, 70%] in 2004 in the three sites, respectively (Fig 5B).

In addition to the intrinsic transmissibility of the etiologic agent, the basic reproductive number is also determined by the contact pattern of the host population. To disaggregate these two factors, our model-inference system explicitly accounts for the latter, which further includes differential contacts among different age groups (Eq 4) as well as imperfect-mixing among the susceptibles and infectious (represented by the mixing parameters *m*_*1*_ and *m*_*2*_ in Eq 1). During our study period, estimates for contacts among age groups (*β*_*2*_ to *β*_*6*_ in the contact matrix; Eq 4), while varied slightly from year to year, did not exhibit a clear secular trend and were similar among the three sites (S1 Fig). In contrast, we found that the mixing parameter *m*_*1*_ (i.e., the exponent of the susceptibles in Eq 1) decreased over time with increased vaccination coverage; and similar temporal pattern was estimated for all three sites (Fig 5C). Similarly, we found that the mixing parameter *m*_*2*_ (the exponent of the infectious in Eq 1) decreased over time; this decrease was most substantial in Beijing (Fig 5D). In addition, *m*_*2*_ was estimated to be lower in Shandong than Beijing. This may explain the milder outbreaks in Shandong in recent years despite the higher population susceptibility therein (Fig 4A).

Previous studies have identified mixing in schools as a key factor driving the rise of measles cases following school opening in the fall during the pre-vaccine era [14,17,27]. Here we modeled this effect using a school term-time forcing function controlled by the amplitude of forcing (*b*_*1*_; see Eq 3) and school schedules. Interestingly, the estimated forcing amplitude was higher for Beijing (~0.9) than Guangzhou and Shandong (~0.6 for both locations; Fig 5E), which is consistent with the greater school enrollment rates in Beijing [28]. In addition, to recreate the observed seasonal patterns, a second seasonal forcing was needed. The estimated seasonal amplitude (*b*.*season*; see Eq 2) was highest in Beijing (mean = 0.76), moderate in Shandong (0.50), and lowest in Guangzhou (0.30; Fig 5F).

## Discussion

Much of previous measles research has focused on industrialized countries. To date, the transmission dynamics of measles in China, where the world's largest population resides, remain largely unknown. Here we have developed a comprehensive model-inference system that takes into account complex population demographics, contact patterns, mass vaccination, and under-reporting. When fitted to only yearly incidence data for the entire population using parameter priors selected based on quasi-decadal monthly incidence, our model is able to accurately estimate out-of-sample age-specific epidemic data. Using this validated model-inference system, we are thus able to reveal epidemiological and demographical characteristics key to measles transmission during 1951–2004 in 3 key locations in China. These characteristics include age-specific population susceptibility and incidence rates, the basic reproductive number (*R*_*0*_), reporting rate, the importance of school mixing, and amplitude of seasonality.

The basic reproductive number (*R*_*0*_) for measles is of importance as it is used to inform target vaccination levels for measles elimination. However, a recent systematic review [29] revealed a large discrepancy in *R*_*0*_ estimates (58 estimates ranging from 1.43 to 77.38) and none were estimated for China. Here we estimate that *R*_*0*_ for Beijing, Guangzhou, and Shandong—two major cities and one province in China—was around 16, and stayed at similar levels in the pre- and post-vaccine eras (Fig 5A). Based on our estimate, to eliminate measles in these locations, a minimal population herd immunity of 93.8%, or with a vaccine efficacy (VE) of 95% [30], a minimal vaccine coverage of 98.7% [*V*_*T*_ = (1–1/*R*_*0*_) ÷ VE = (1–1/16) ÷ 0.95] is needed. Note the latter, more conservative estimate is above the targeted 95% vaccination threshold [1].

The stable estimation of *R*_*0*_ here is due to careful control of changes in case reporting and population mixing pattern over time. We found that reporting rates have increased substantially in all three locations over the five decades, consistent with the enhanced disease surveillance in China. In addition, we also found that the intensity of mixing, as represented by the mixing parameters *m*_*1*_ and *m*_*2*_, tended to decrease with increased vaccination coverage. These changes conform with the intuition that vaccination can provide indirect protection to the entire population, i.e. herd immunity. In contrast, our model-inference system did not identify significant changes in the contact parameters (*β*_*2–6*_) over time. Such changes could be masked by the wide ranges of our posterior estimates (S1C–S1G Fig) as age-specific data were not available for the entire study period to constrain these age-related model parameters. Nonetheless, the accurate estimates of independent, out-of-sample age-grouped incidence over 20 years for both Beijing (Fig 3A, 1^st^ panel) and Shandong (Fig 3C) indicate our *β* estimates are broadly accurate.

Population susceptibility, i.e. the complement of herd immunity, reflects the vulnerability of the population to infection. This variable is commonly measured by serological surveys. However, such studies are limited by the small number of people surveyed (e.g. typically in the hundreds). Here using a model-inference system, we are able to estimate population susceptibility by age group in weekly intervals (Fig 4). The estimates reveal that population susceptibility has remained low in Beijing and Guangzhou due to high vaccination rates but has increased substantially in Shandong, particularly in children and young adults.

This differential increase in susceptibility across locations may have profound public health implications for current measles epidemic dynamics in China. In a recent study [5], we found that large industrial cities in China with large migrant populations supported endemic measles transmission during 2005–2014. Both Beijing and Guangzhou were among such cities; for instance, in 2010, 35.7% (7.0/19.6 millions) of Beijing's population were migrants, of which 8.5% came from Shandong (census data [31]). These migrants, likely from regions with higher susceptibility as in Shandong, could be subject to greater risk of infection. With an *R*_*0*_ of 16, a city of 35.7% migrants (assuming 10% and 5% susceptibility for migrants and local residents, respectively) would have an effective reproductive number (*R*_*e*_) of 1.09, slightly above unity and thus capable of sustaining an epidemic. This simple assessment suggests that migrants may have been (and continue to be) a vulnerable subpopulation and contributed to the persistent transmission of measles in big cities despite high vaccination coverage therein. In addition, this finding suggests that catch-up vaccination targeting migrant populations might be an efficient means of controlling current epidemics in these big cities. Indeed, such targeted catch-up vaccination has been implemented in Beijing since 2005 (e.g., ~2 M migrant workers were vaccinated during 2005–2010 [32]) and substantially reduced the number of infections in migrant workers in recent years [32].

Our study also reveals interesting differences in measles seasonality among the three study locations. As found previously for industrialized countries [14,17], increased mixing among school-age children during school terms can facilitate measles transmission. Among the three sites, the estimated amplitude of school forcing was highest in Beijing, the capital and cultural center of China. However, additional seasonal forcing was needed to reproduce observed seasonal epidemic patterns. The estimated seasonal amplitude decreased with decreasing latitudes in the three sites (Fig 1A). This finding suggests that climate condition may also play a role in measles seasonality. More specifically, winter indoor heating in cold climates (e.g., Beijing and Shandong in this study) may increase crowding and/or reduce ventilation and hence increase the risk of infection during cold months. In comparison, there is no indoor heating in Guangzhou due to its mild winters.

We recognize a number of limitations in our study. First, due to a lack of long-term city-level data, we used Shandong province as a "control" for the two study cities (i.e. Beijing and Guangzhou). The aggregate data for Shandong from its many cities (17; as of 2018) may have masked some of the local characteristics. In addition, we note that all three sites are located in the more developed coastal region of China and thus may be less representative of inland regions. Second, synthetic testing indicates that parameter collinearity exists and may reduce the identifiability of certain parameters in our inference system. In particular, we found that overestimation of *R*_*0*_ can be compensated by underestimation of the mixing parameter *m*_*2*_ or *vice versa*. However, this issue is relatively mild. In addition, out-of-sample data (e.g., age-specific data) can be used to validate model estimates, which was done here. Third, due to a lack of data, in the model we assumed that migrants have the same susceptibility as local residents. This may lead to underestimation of the population susceptibility in Beijing and Guangzhou after the mid-1990s, when migrants started to account for >10% of the total population. More in-depth analyses of this issue based on the findings presented here are underway. Fourth, for simplicity, we assumed the same reporting rate for all age groups. In reality, reporting rates for infants and young children are likely higher than older age groups. As a result, our model-inference system tended to underestimate incidence in infants and overestimate incidence in adults >50 yrs (Fig 3C). Fifth, we did not include supplementary immunization activities (SIAs) in our model. However, a recent study [26] has evaluated the impact of SIAs on reducing population susceptibility in six provinces in China (including Shandong) and found that efficiencies of SIAs prior to 2005 (<50%) were lower than later years (32–87%). Lastly, for simplicity, we assumed the duration of maternal immunity follows an exponential distribution with a mean of 6 months. Recent studies [32–34] have suggested that infants born to mothers immunized by vaccination may have weaker and shorter passive immunity relative to the pre-vaccine era, and thus are subject to risk of infection earlier in life. Future work will test such an impact using more detailed recent data.

In summary, we have developed a model-inference system capable of inferring the underlying transmission dynamics of measles in China, based on sparse observations. Fitted to highly aggregated incidence data, the model-inference system is able to estimate population susceptibility, the basic reproductive number (*R*_*0*_) and other key epidemiological parameters during 1951–2004, a period that spans the pre-vaccine and modern mass-vaccination eras. Our findings reveal population and epidemiological characteristics crucial to understanding the current persistence of measles epidemics in China and for devising future elimination strategies.

## Methods and materials

### Data

Yearly data on demographics (i.e., birthrates, death rates, and migrations), vaccination coverage and doses, and measles incidence during 1951–2004 reported for Beijing, Guangzhou, and Shandong were used in our model-inference system. These data (Fig 1 and S1 Table) were compiled from many sources or estimated in this study. Compilation of reported data and estimation of missing variables are summarized in S1 Text.

### Epidemic model

The main measles transmission model represents susceptible-exposed-infectious-recovered (SEIR) dynamics with 4-age groups (i.e. <1, 1–14, 15–50, and >50 yr olds) per Eq 1:

$$\{\begin{array}{c} \begin{array}{c} \frac{dS_{i}}{dt}=-\sum_{j=1}^{4}\beta_{ij}\left(t\right)S_{i}^{m_{1}}I_{j}^{m_{2}}/N_{j}\\ \frac{dE_{i}}{dt}=\sum_{j=1}^{4}\beta_{ij}\left(t\right)S_{i}^{m_{1}}I_{j}^{m_{2}}/N_{j}-\frac{E_{i}}{Z}+\alpha_{i}\left(t\right)\\ \frac{dI_{i}}{dt}=\frac{E_{i}}{Z}-\frac{I_{i}}{D} \end{array}\\ \frac{dR_{i}}{dt}=\frac{I_{i}}{D}\\ \frac{dM}{dt}=B-\frac{M}{180} \end{array}$$ (Eq 1)

where *S*_*i*_, *E*_*i*_, *I*_*i*_, *R*_*i*_ and *N*_*i*_ are, respectively, the numbers of susceptible, exposed (i.e. latently infected), infectious, recovered people and population size in the *i*-th age group; *B* is the number of newborns with maternal immunity (see calculation details and other demographic processes at the end of this section) and *M* is the number of infants with maternal immunity (note, we assume a mean maternal immunity period of 180 days); *t* is time in days. The exponents *m*_*1*_ and *m*_*2*_ describe the level of inhomogeneous mixing [35,36]; *Z* and *D* are the latent and infectious period, respectively. *α*_*i*_(*t*) is the number of travel-related infections (i.e. seeding) in the *i*-th group on day *t*; it was set to 1 case in Groups 1–3 during two major holidays in China: the national day on Oct 1 and the Chinese New Year in Jan/Feb. This seeding allows reintroduction of measles after local epidemic extinction.

The transmission rate at time *t* (day of the year here), *β*_*ij*_(*t*), varies with an annual cycle per:

$$\beta_{ij}\left(t\right)=\beta_{ij}\{1+b.season\cdot \cos[\frac{2\pi }{365}\left(t-23\right)]\}$$ (Eq 2)

where *β*_*ij*_ is the annual mean transmission rate from the *j*-th to the *i*-th group and *b*.*season* is the amplitude of seasonality. Note that we shift the phase by 23 days to better match observed seasonality. In addition, to capture changes in mixing among school-age children, an additional school term-time forcing function is applied to Group 2 (i.e. 1–14 yr olds), such that

$$\beta_{22}\left(t\right)=\frac{\beta_{22}}{b_{Term}}\cdot \left[1+b_{1}Term\left(t\right)\right]\cdot \{1+b.season\cdot \cos[\frac{2\pi }{365}\left(t-23\right)]\}$$ (Eq 3)

where *b*_*1*_ is the amplitude of school forcing, and *Term*(*t*) is set to 1 for school terms, -1 for summer breaks, and 0.5 for winter breaks (note that winter breaks in China last for 5 weeks spanning the Chinese New Year when mixing tends to be higher) [17]. Per [17], we adjust $\beta_{22}$ by dividing the mean forcing, i.e. $b_{Term}$, such that the school forcing averages to 1 over a year.

For a 4-age group model, the ***β*** matrix includes 16 elements. To reduce the number of parameters, we formulate ***β*** using 6 parameters as follows:

$$\beta =\left[\begin{array}{cccc} \beta_{1} & \beta_{1} & \beta_{5} & \beta_{4}\\ \beta_{1} & \beta_{2} & \beta_{6} & \beta_{4}\\ \beta_{5} & \beta_{6} & \beta_{3} & \beta_{4}\\ \beta_{4} & \beta_{4} & \beta_{4} & \beta_{4} \end{array}\right]$$ (Eq 4)

where, *β*_*1*_ *to β*_*4*_ represent within-group contact for the four age groups, respectively; *β*_*5*_ (*β*_*6*_) represents mixing between infants (children) and parents. As contact with the elderly tends to be less frequent than other age groups, we set all those related to Group 4 (>50 yr) to *β*_*4*_. Further, for better configuration of the priors, we set *β*_*1*_ to 1 and estimate the relative magnitude of *β*_*2*_–*β*_*6*_ (see S1 Text for details). The absolute values of ***β*** are then determined by the basic reproductive number (*R*_*0*_) via the relationship [37]:

$$R_{0}={\text{eigen}}_{\text{max}}\left(n\beta D\right)$$ (Eq 5)

where eigen_max_(·) denotes the function giving the maximum eigenvalue of a matrix, and ***n*** is a diagonal matrix with elements *n*_*i*_ = *N*_*i*_/∑*N*_*i*_ (*i* = 1, …, 4), i.e. the fraction of population in Group-*i*.

We then superimposed demographic processes onto the transmission model (Eq 1). These include birth, death, aging, migration, and vaccination based on population and vaccination data reported for each year during 1951–2004 (S1 Table and S1 Text). All processes were updated in daily time step. Briefly, for the birth process, newborns with maternal immunity (i.e., *B*) were added to compartment *M* (Eq 1, 5^th^ line) and the remainder were added to compartment *S*_*1*_ (i.e., susceptibles aged <1 yr). For simplicity, we roughly estimated the proportion of newborns with maternal immunity as 1–1/*R*_*0*_ ≈ 1–1/15 = 93.3%; that is, the complement of long-term equilibrium susceptibility [3,37], assuming *R*_*0*_ = 15 (i.e., the mid-point of the reported 12–18 range [3]). Note that since maternal immunity wanes quickly (here the mean sojourn time in compartment *M* was 6 months), for *R*_*0*_ values slightly different from 15, this approximation would only cause a slight shift in timing for a small number of newborns to enter the susceptible pool (i.e., *S*_*1*_). Daily age-specific deaths were subtracted from the corresponding age groups; for simplicity, the same death rate was used for all disease classes from the same age group. Aging was modeled as an exponential process. To include the migrant population, daily net numbers of age-specific migrants, assumed to have the same susceptibility and infection rate as the locals, were added to the corresponding age groups. For simplicity, vaccination was “administrated” in the model at 1-yr of age and with 1 "effective" dose. Nevertheless, changes in number of vaccine doses and vaccine efficacy over time were accounted for in our immunization rate estimates, which were used as model input of the "effective" vaccine coverage.

### Estimation of model state variables and parameters

We applied a modified particle filter [38,39] jointly with the model described above and the yearly incidence data to estimate the state variables (i.e. *S*_*i*_, *E*_*i*_, *I*_*i*_, *R*_*i*_, and *M*; *i* = 1,…, 4) and parameters (*D*, *Z*, *m*_*1*_, *m*_*2*_, *b*_*1*_, *b*.*season*, *R*_*0*_, and *β*_*2*_–*β*_*6*_). Briefly, we first initiated a model ensemble using a suite of random state variables and parameters (*n* = 5000 model replicates, or particles) and ran the model stochastically with a daily time step from 1851 for 100 years to reach equilibrium. Beginning in 1951 (when incidence data became available), we ran the model in conjunction with a particle filter [38,39] to incorporate the data and estimate the model state. This filtering process was done sequentially by repeated prediction-update cycles. In each cycle (i.e. each year here), the model ensemble (i.e. the 5000 particles) was integrated forward in time for a year per the model (this generates the prediction). To update the model state, model-estimated incidence was aggregated for the year, adjusted by reporting rate for that year (estimated simultaneously by the filter), and used to compute the likelihood of each particle, as compared to the observation (i.e. yearly incidence). The posterior of model state was then computed using Bayes' rule [38,39] at the end of each year. See S1 Text for details in choices of priors for the parameters.

As mentioned in the Introduction, many factors shaping the epidemic dynamics of measles in China have changed dramatically during our study period (1951–2004). These changes create challenges for the filter system, which tends to converge to a small sub-state-space and be trapped there (i.e. an issue termed particle impoverishment [38,40]). To rejuvenate the model-inference system, here we applied space re-probing, a technique that allows the filter to continuously explore the full state-space and thus adapt to changes over time [24,25]. To account for stochasticity, we repeated each model-inference simulation 10 times and combined the estimates (mean and standard deviation) per Rubin's rules [41,42].

### Selection of optimal parameter priors

The model-inference system estimates 13 parameters simultaneously based on 54 yearly incidence records from 1951 to 2004. To improve filter performance, for 5 of the more sensitive parameters (i.e. *R*_*0*_, *m*_*2*_, reporting rate, *b*_*1*_, and *b*.*season;* see S1 Text for detail), we parsed the parameter space into segments of subspace, ran the model-inference system using all possible combinations of subspace, and selected the combination generating the best fit (based on likelihood, correlation, and root mean square error) to the yearly incidence and available quasi-decadal monthly incidence. This process was carried out by 2 rounds of selection, for each study location. First, for each parameter, the full prior range was parsed into mutually exclusive segments; for instance, for *b*.*season* (the amplitude of seasonality), the full range [0, 1) was parsed into: [0, 0.2), $\left[0.2,\;0.4\right)$, ….,  [0.8, 1). All possible combinations were tested (270 combinations x 5 repeated runs for each to account for stochasticity), generating 5–10 top combinations. In the second, neighboring space of the top combinations from Round 1 were tested; for instance, if $\left[0.4,\;0.6\right)$ was identified as the best subspace for *b*.*season* in Round 1, then $\left[0.3,\;0.5\right),\;\left[0.5,\;0.7\right)$, and $\left[0.3,\;0.7\right)$ were tested in the second round. The best prior combination was then selected for each study site and used in the final model-inference. The specific prior ranges tested are discussed in S1 Text and the selected final "optimal" priors are shown in Figs 5 and S1.

### Validation of the model-inference system using synthetic testing

We validated the model-inference system and the prior selection strategy using synthetic testing. The basic idea is that, if the method is effective, it should be able to recover the true state variables and parameters used to generate the mock epidemic. Four mock, or synthetic, epidemics were generated for this test, using the model with population data for Beijing and 4 different combinations of *b*_*1*_ and *b*.*season*: *b*_*1*_ = 0.9 with *b*.*season* = 0.3, 0.5, or 0.75 and *b*_*1*_ = 0.6 with *b*.*season* = 0.75. The values for other parameters used here are shown in S3–S6 Figs. As the model was run stochastically, we aggregated 5000 simulations to generate each synthetic dataset (i.e. the "truth"). To mimic available observations, model generated incidence was aggregated over all age-groups in yearly intervals and quasi-decadal monthly incidence was aggregated for 1951–1966, 1967–1978, 1979–1984, and 1985–1996. Differences among the 4 synthetic datasets are shown in S2 Fig. For each synthetic epidemic, the quasi-decadal monthly incidence was first used to select the optimal priors, and then with these priors, the model-inference system was used to construct the underlying transmission dynamics based on the synthetic yearly incidence for the entire population. The final estimates of state variables and parameters were then compared to the truth.

## Supporting information

S1 Text Description on the study sites, measles and demographic data, and supplementary information on the formulation of the measles model-inference system. (DOCX)

S1 Table Summary of data type and source. (DOCX)

S2 Table Performance of the model-inference system using synthetic data. (DOCX)

S1 Fig Estimates of model parameters for the three locations during 1951–2004, not shown in Fig 5 in the main text: infectious period (A), latent period (B), and *β*_*2*_ to *β*_*6*_ (C-G). Solid lines show the mean posterior estimates and shaded areas show the 95% CIs; thick dashed lines show the prior ranges and thin dashed lines show the mean values of the priors.
(TIF)

S2 Fig Synthetic truths. (A) Yearly incidence generated by the model using different combinations of *b*_*1*_ and *b*.*season*. Solid lines show incidence for the entire population; these data were used as "observations" in the synthetic testing. Dashed lines show incidence for Group 2 (i.e. 1–14 yr olds). Monthly incidence aggregated for 1951–1966 (B), 1967–1978 (C), 1979–1984 (D), 1985–1996 (E), and 1997–2004 (F) were used for selection of the optimal priors.
(TIF)

S3 Fig Results of synthetic testing of the model-inference system, using synthetic truth 1. (A) Model-fits to the observations (i.e. yearly incidence for the entire population). (B) Model estimates of incidence in the key age group (i.e. 1–14 yr olds); note these age-specific ‘truths’ were not used in model fitting. Model fits to monthly incidence aggregated for 1951–1966 (C), 1967–1978 (D), 1979–1984 (E), and 1985–1996 (F). These monthly aggregates were not directly used for model-fitting, but used to select the optimal parameter priors. Model estimates of *weekly* incidence for the entire population (G) and 1–14 yr olds (H), compared to the truth (not used for model-fitting). Estimates of key model parameters compared to the truth: *R*_*0*_ (I), *m*_*2*_ (J), reporting rate (K), *b*_*1*_ (L), *b*.*season* (M) and *β*_*2*_ to *β*_*6*_ (N-R).
(TIF)

S4 Fig Results of synthetic testing of the model-inference system, using synthetic truth 2. (A) Model-fits to the observations (i.e. yearly incidence for the entire population). (B) Model estimates of incidence in the key age group (i.e. 1–14 yr olds); note these age-specific ‘truths’ were not used in model fitting. Model fits to monthly incidence aggregated for 1951–1966 (C), 1967–1978 (D), 1979–1984 (E), and 1985–1996 (F). These monthly aggregates were not directly used for model-fitting, but used to select the optimal parameter priors. Model estimates of *weekly* incidence for the entire population (G) and 1–14 yr olds (H), compared to the truth (not used for model-fitting). Estimates of key model parameters compared to the truth: *R*_*0*_ (I), *m*_*2*_ (J), reporting rate (K), *b*_*1*_ (L), *b*.*season* (M) and *β*_*2*_ to *β*_*6*_ (N-R).
(TIF)

S5 Fig Results of synthetic testing of the model-inference system, using synthetic truth 3. (A) Model-fits to the observations (i.e. yearly incidence for the entire population). (B) Model estimates of incidence in the key age group (i.e. 1–14 yr olds); note these age-specific ‘truths’ were not used in model fitting. Model fits to monthly incidence aggregated for 1951–1966 (C), 1967–1978 (D), 1979–1984 (E), and 1985–1996 (F). These monthly aggregates were not directly used for model-fitting, but used to select the optimal parameter priors. Model estimates of *weekly* incidence for the entire population (G) and 1–14 yr olds (H), compared to the truth (not used for model-fitting). Estimates of key model parameters compared to the truth: *R*_*0*_ (I), *m*_*2*_ (J), reporting rate (K), *b*_*1*_ (L), *b*.*season* (M) and *β*_*2*_ to *β*_*6*_ (N-R).
(TIF)

S6 Fig Results of synthetic testing of the model-inference system, using synthetic truth 4. (A) Model-fits to the observations (i.e. yearly incidence for the entire population). (B) Model estimates of incidence in the key age group (i.e. 1–14 yr olds); note these age-specific ‘truths’ were not used in model fitting. Model fits to monthly incidence aggregated for 1951–1966 (C), 1967–1978 (D), 1979–1984 (E), and 1985–1996 (F). These monthly aggregates were not directly used for model-fitting, but used to select the optimal parameter priors. Model estimates of *weekly* incidence for the entire population (G) and 1–14 yr olds (H), compared to the truth (not used for model-fitting). Estimates of key model parameters compared to the truth: *R*_*0*_ (I), *m*_*2*_ (J), reporting rate (K), *b*_*1*_ (L), *b*.*season* (M) and *β*_*2*_ to *β*_*6*_ (N-R).
(TIF)
