# Supplementary material for: Characteristics of measles epidemics in China (1951-2004) and implications for elimination: A case study of three key locations
Source: PLoS Comput Biol. 2019 Feb 4;15(2):e1006806. doi: 10.1371/journal.pcbi.1006806 (PMC6375639; doi:10.1371/journal.pcbi.1006806)
Supplement: S1 Text — (DOCX) [file pcbi.1006806.s001.docx]

Supporting Information

**Characteristics of Measles Epidemics in China (1951-2004) and Implications for Elimination: A Case Study of Three Key Locations**

Wan Yang, Juan Li, Jeffrey Shaman

In this document, we present 1) A brief description of the three study sites; 2) Details of the measles and demographic data used in our model-inference system; and 3) Supplementary information on the formulation of the measles model-inference system.

**1. Study locations**

In this study, we model the transmission dynamics of measles during 1951-2004 in three locations in China—Beijing, Guangzhou, and Shandong. Beijing is the capital of China. The city is located in Northern China (39°55'N, 116°23'E) and has a humid continental climate with hot summers (ca. Jun-Aug, mean temperature: 24°C) and long, cold winters (ca. Nov-March, Mean temperature: –1°C)[1]. Public heating, which has been provided to all residences since the 1950s, starts on Nov 15^th^ and ends on March 15^th^ every year. Total population size was 2.8 M in 1953 (1^st^ census), 9.2 M in 1982 (3^rd^ census), and 19.6 M in 2010 (6^th^ census)[2]. Note that the dramatic population increase in the early years was in part due to the expansion of the administrative region—Beijing increased from 3600 km^2^ in 1953 to 16,411 km^2^ in 1982 and since has remained the same [2,3]; in later years, the population increase was largely due to the influx of migrants (Fig 1 in the main text).

Guangzhou is the capital of Guangdong province. The city is located in southeastern China (23°08'N, 113°16'E) and has a humid subtropical climate with long, hot summers (ca. April-Sep/Oct, mean temperature: 27°C) and short, mild winters (ca. Dec-Jan, mean temperature: 14°C)[4]. Total population size was 2.8 M in 1953 (1^st^ census), 5.2 M in 1982 (3^rd^ census), and 12.7 M in 2010 (6^th^ census)[5]. Guangzhou has been, and continues to be, an economic center in southern China.

Shandong is a province located in eastern China (between 34°23'-38°24'N and 114°48'-122°42'E). Shandong has a temperate climate; winters are roughly from Dec to Feb, with mean temperatures ranging from –4°C to 4°C depending on geolocation [6]. Public residential heating is provided in most cities, and as in Beijing, typically starts on Nov 15^th^ and ends on March 15^th^. Total population size was 18.9 M in 1953 (1^st^ census), 74.4 M in 1982 (3^rd^ census), and 95.8 M in 2010 (6^th^ census)[7]. The 4-fold increase in population during 1953-1982 was largely due to high birthrates at the time (Fig 1).

**2. Measles epidemiological and demographic data.**

Below we describe the compilation of measles epidemiological data and related demographic data for each of the three study locations (S1 Table). In addition, we detail the methods used to estimate missing data. As measles epidemiological data, including incidence and vaccination, tended to come from the same source, we organize the subsections by location, as opposed to data type. Shared data (e.g. infant mortality rates) used for all locations are presented at the end.

**2.1. Data for Beijing.**

2.1.1. Measles data. Measles data for Beijing were taken from Li et al. 2013 [8](hereafter referred to as Li2013). These include: 1) yearly incidence (1951-2004) for the entire population (Fig 2 thereof); 2) yearly incidence (1978-2004) for 1-14 yr olds (Fig 6 thereof); 3) age-specific incidence (in particular, for <1 yr olds; Fig thereof); 4) average monthly incidence aggregated for 1985-1996 and 1997-2004 (Fig 5 thereof); and 5) measles vaccination coverage for the 1^st^ dose from 1971 to 2004 and for the 2^nd^ dose from 1984 to 2004 (Fig 6 thereof). These data were reported in the form of figures. To obtain the numbers, we digitized the figures using the “digitizeR” package in R (https://github.com/ankitrohatgi/digitizeR). The same digitization software was used to extract data from figures in other instances in this study.

2.1.2. Estimation of immunization rates. Based on descriptions in Li2013, a 1-dose measles vaccination program was implemented in Beijing starting in 1966; however, vaccination coverage before 1971 was not reported. To estimate vaccination coverage during 1966-1970, we used the median value reported for 1971-1973, as no clear time trend were observed for those early years. To account for vaccine efficacy (VE), we multiplied the vaccination coverage by 0.8 for years with 1-dose measles vaccination and 0.95 for years with 2-dose measles vaccination and refer to this combined estimate as the immunization rate. Note that, these VE values are more conservative compared to those reported in China, i.e. >85% for 1-dose and >95% for 2-doses [9,10]. The combined estimates are shown in Fig 1 in the main text.

2.1.3. Demographic data. In China, a household registration system (called "Hukou")[11] records information on births, deaths, marriages, divorces, and permanent movements of all local residents (i.e. those with a Hukou issued by the city in which they reside) for each city. As such, the population registered in the Hukou system reflects the size of the local resident population, whereas the total population in the city includes both locals and migrants who do not hold a Hukou issued by that city. For Beijing, population size for local, migrants, and the total population, respectively, were compiled from three sources: 1) The Information Center of Beijing Municipal Commission of Health and Family Planning reported the numbers of births, deaths, local residents from 1949 to 2015; however data before 2000 were only reported in 5-yr intervals [12]; 2) Beijing Bureau of Statistics (BJ-BoS) reported the local resident population, permanent migrant population (defined by BJ-BoS as migrants who live in the city for more than 6 months), birthrates, death rates from 1978-2014; 3) Li2013 reported the number of measles cases as well as per capita measles incidence during 1951-2011, from which the total population size for each year can be back-calculated. Comparison of overlapping data showed consistent reporting from these three sources. We thus combined all datasets to obtain yearly estimates of local, migrant, and total population, as well as birthrate and death rate for our study period (1951-2004).

**2.2 Data for Guangzhou.**

2.2.1 Measles data. Yearly incidence data during 1951-2004 for the entire population in Guangzhou were taken from Fig 1 in Yang et al. 2014 [10]. While the paper also reported incidence for each month of the year (Fig 2 thereof), these data were aggregated for 5 decades (1965-2012) and thus likely did not reflect the true seasonality of measles during different time periods. To our knowledge, no other data detailing the seasonal pattern of measles are available for Guangzhou at this writing. Therefore, we instead used reports on regions closely related to Guangzhou. Specifically, two seasonal datasets were used: 1) monthly data for Guangdong (i.e. the province encompassing Guangzhou), aggregated for 1951-1966, 1967-1978, 1979-1986, and 1987-2000 from Table 1 of Lin et al. 2002 [13]; and 2) monthly data for Tianhe (one of the districts in Guangzhou) aggregated for 1985-2001 from Table 1 of Tian et al. 2003 [14].

2.2.2. Estimation of immunization rates. The Guangzhou City Bureau of Statistics (GZC-BoS) reported measles vaccination rates for 1990, 1995, and all years from 1998 onwards [5]. These reports only provide one overall estimate without specifying the number of vaccine doses. In addition, Jin & Chen 1997 [15] reported vaccination rates among <1 yr olds (i.e. 1^st^ dose measles vaccine) for Years 1986-1995 (Table 2 thereof). Cross-checking the two datasets suggests that the GZC-BoS data were vaccination rates of the 1^st^ vaccine dose. We thus combined the two datasets to obtain the 1^st^ dose vaccination rates from 1986 to 2004. During 1968-1985, measles vaccination was administrated in Guangzhou [16], but data were not available. We thus modeled the differences between vaccination rates in Beijing and Guangzhou in years data were available for both cities (i.e. 1986-2011) as a logistic function (based on the temporal trend) and used the fitted differences in coverage and estimates for Beijing during 1966-1985 to estimate the coverage in Guangzhou in those years. Assuming the gap in coverage for the 2^nd^ dose between the two cities followed the same logistic function as for the 1^st^ dose, the 2^nd^ dose coverage in Guangzhou was then estimated by subtracting the coverage in Beijing by the estimated gap. Immunization rates combining 2 vaccine doses were then estimated using the same vaccine efficacies used for Beijing.

2.2.3. Demographic data. The GZC-BoS reported registered (i.e. local) population size, birthrate, and death rate in Guangzhou from 1949 onwards. However, information on migrant or total population is incomplete. Migration data for the registered population (i.e. emigration of locals and immigrants with a Hukou issued by the city) were recorded for 1962-1990 in ~5 yr intervals, and for 1991-1998 in yearly intervals. In addition, total population size (i.e. locals + migrants) was recorded in three of the five censuses during our study period (i.e. 1982, 1990, and 2000). Fortunately, migrant population size from 1989 to 2006 were recorded in the city's Statistical Yearbooks (in print) and reported in Yao et al. 2009 [17]. We thus estimated total population size for those years by adding the migrant population to the registered population.   For the period 1962-1980 where migration was nominal, we used the migration data reported by GZC-BoS to compute the total population size.  For years before 1962 when no data were available and migration was nominal, we set the total population size to the registered population size.

**2.3. Data for Shandong.**

2.3.1. Measles data. Yearly incidence data during 1951-2004 for the entire population were compiled from two journal articles: 1) Xu et al. 1995 [18] which reported incidence during 1951-1994 (Fig 1 thereof; referred to as Xu1995 hereafter) and 2) Li et al. 2012 [19] which reported incidence during 1963-2005 (Fig 1 thereof). Records from these two data sources for overlapping years (i.e. 1963-1994) were consistent. In addition, Xu1995 also reported monthly aggregates for 1951-1966, 1967-1978, 1979-1989, and 1990-1994 (Table 1 thereof). Further, a recent study [20] reported detailed age-specific incidence data for Shandong during 1985-2011; these data were used to test the accuracy of our age-grouped incidence estimates (Fig 3C in the main text).

2.3.2. Estimation of immunization rates and validation. An extensive search on vaccination coverage in Shandong showed that no detailed data were available. We thus resolved to estimating the vaccination coverage based on the total number of vaccine doses used each year during 1967-1994 reported in Xu1995, using a multistep routine. First, we adjusted the yearly vaccine usage for population size by dividing the total number of newborns in the previous 4 years (the 4-yr period was chosen by trial and error). This adjustment is needed because the raw numbers were much higher than would be expected, and at best reflected the temporal trend in vaccination coverage. For example, according to Xu1995, an average of 7.26 million person-doses were used each year during 1967-1994 and most were administrated to infants <1 yr; in comparison, the number of newborns per year ranged from 0.84 to 2.29 million with a mean of 1.42 million during the same time period. Second, we assumed a 5% coverage in 1967 and 85% coverage in 1985 (based on descriptions in Xu1995), and linearly mapped the adjusted vaccine usage to vaccination coverage. Third, to account for increases in vaccine efficacy over time, in particular, due to adoption of cold chain systems from 1985 onwards, we further adjusted the estimates from Step 2 for years with the cold chain by a factor of 1.1 to 1.3 with a yearly increase of 0.025. Fourth, we regressed the estimates for 1967-1994 on the year, and extrapolated the regression model to estimate the vaccination coverage for years without vaccine usage data (i.e. 1995-2004).

As the vaccine usage data from Xu1995 were mostly for <1 yr olds, the vaccination coverage estimates described above only represent coverage for the 1^st^ vaccine dose. Two-dose vaccination was administrated from 1986 onwards nationwide. To estimate coverage for the 2^nd^ vaccine dose, we first computed the difference in the 1^st^ dose coverage between Beijing and Shandong and regressed this coverage difference on time (in year) to estimate the gap in coverage between the two locations. Assuming the gap in coverage for the 2^nd^ dose between the two locations changed at the same rate, the 2^nd^ dose coverage in Shandong was then estimated by subtracting the coverage in Beijing by the estimated difference. The combined immunization rate, adjusting for vaccine efficacy and vaccine dose, was then computed using the same method described for Beijing. As a simple validation, our estimates of average immunization rate were 20% during 1967-1978, 77% during 1979-1997, and 85% during 1998-2004, which were close to the numbers mentioned in Li et al. 2012 (i.e. 20%, 80%, and 90% for the three periods, respectively)[19].

2.3.3. Demographic data. The Shandong Provincial Bureau of Statistics [7] reported local and total population size, birthrate and death rate for most years since 1949. The only missing data were for years 1950 and 1951, which were estimated using linear interpolation. As for Beijing and Guangzhou, migrant population size was computed as the total minus local population.

**2.4. Shared data for all locations.**

Infant mortality rates during 1950-2015 (in yearly intervals) were obtained from [21]. Death rates for 1-14, 15-50, and >50 yr olds were computed based on age-specific mortality data from the 1982 and 2000 censuses [2]. Age structure of migrants, i.e., the percentages of migrants who are <1, 1-14, 15-50, or >50 yr (the four age groups in our measles transmission model), was estimated based on census data for Beijing in 2010 [22]. Note that, while these data were reported for 2010 (i.e., outside our study period of 1951-2004), the age structure of migrants did not change substantially compared to earlier years (e.g. 1997 and 2001 [23]), for which data were less complete.

**3. Supplementary information on the measles model-inference system**

Previous studies have shown that increased mixing among school-age children during school terms was a leading factor for the Oct-to-July epidemics in industrialized countries in the pre-vaccine era and that, an age-structured susceptible-exposed-infected-removed (SEIR) model capturing this heterogeneous mixing was able to reproduce observed epidemics [24-28]. Given this mechanism and available data, we used a four-age-group SEIR model (<1, 1-14, 15-50, and >51 yr) to parsimoniously capture maternal immunity, vaccination, differential mixing among age groups, imperfect mixing within groups, and seasonality (Fig 2 and Methods in the main text). These age group divisions were chosen based on the education system in China (≤~15 yr olds are children provided with free compulsory education), availability of demographic data (typically aggregated for <1 and 1-14 yr olds), availability of measles surveillance data (in Li2013, incidence was aggregated for 1-14 yr olds as they were the main affected age group), vaccination schedule (1^st^ dose at 8-months of age and 2^nd^ dose at 18-months of age), and the age structure of migrants (~90% are ≤50 yr olds). The model per Eqn 1 is described in the main text. Below, we provide further details in the setting and testing of parameters:

1. *m_1_*: the exponent of the susceptibles (representing imperfect mixing). Since vaccination can reduce the number of susceptible individuals in the population, the level of mixing among the susceptibles would likely decrease with increased vaccination coverage. Indeed, in our preliminary testing of the model-inference system, we found that simulations capable of recreating the observed yearly incidence curve tended to be those with *m_1_* decreasing gradually in the post-vaccine years; further, the decrease in *m_1_* resembled a logistic curve. As such, we modeled the change in *m_1_* using a logistic function for years after the introduction of vaccine per: $m_{1}\left( t \right)=m_{1}\left( 0 \right)-\frac{0.12}{1+e^{-0.3(t-t_{c})}}$, where *t* is time in year; *t_c_* is the critical year chosen based on the level of vaccination coverage (in this study, *t_c_* = 1967 for Beijing, 1969 for Guangzhou, and 1977 for Shandong). *m_1_*(0) is the baseline mixing level estimated by the model-inference system along with other parameters, for which a uniform distribution U[0.95, 1] was used as the prior.
2. *m_2_*: the exponent of the infectious (representing imperfect mixing). As public health systems were strengthened over time, isolation of infectious individuals may be timelier in later years, which in turn can reduce the level of mixing among the infectious (*m_2_*). To represent this impact, we tested two forms of *m_2_*. In the first, no specific form was imposed and *m_2_* was estimated along with other parameters by the filter with a prior of U[0.85, 0.95]. In the second, similar to *m_1_*, a logistic function was used: $m_{2}\left( t \right)=m_{2}\left( 0 \right)-\frac{0.05}{1+e^{-0.3\left( t-t_{c} \right)}}$ and *m_2_*(0), the baseline, was estimated by the filter with a prior of U[0.95, 1].
3. Reporting rate (*r*). Little information on the reporting rate of measles cases was available for China, particularly for the early years. To find the proper prior range for *r*, we first estimated the mean reporting rate during 1951-1965 for each of the three locations. To do so, for each city, we ran the model without the filter using different combinations of initial conditions and parameters (except for *r*) randomly drawn using Latin Hypercube Sampling (*n*=5000). Each simulation was run from 1850 to 1965 with the first 100 years discarded. From the 5000 simulations, we then selected those with the same epidemic periodicity as observed. For these runs, we then tested different reporting rates ranging from 5% to 100% with a 5% increment, and selected the one with the maximum likelihood compared to the observed incidence data. Using this method, the preliminary estimates of mean reporting rate during 1951-1965 were 30-40% for Beijing, 20% for Guangzhou, and 16% for Shandong. The prior range of reporting rate for each study site was then centered around the corresponding preliminary estimate. To capture the increase in reporting rate, we imposed a linear increase at a rate of 0.56% per year (i.e. 30% increase over the study period; we also tested a 20% total increase, which did not perform as well). The upper limit of the prior range (from a uniform distribution) was selected using the prior selection strategy, with 3 levels tested for each site: 1) 50%, 60%, and 70% for Beijing; 2) 40%, 50%, and 60% for Guangzhou; and 3) 30%, 40%, and 50% for Shandong.
4. Prior ranges for *R_0_*, the basic reproductive number. Reported *R_0_* for measles is in the range of 12-18 [29]. Therefore, in our prior selection testing, we tested three prior ranges: U[10, 15], [15, 20], and [10, 20].
5. Prior ranges for *β_2_* to *β_6_*. As described in the main text, *β_2_*-*_6_* are the levels of contact relative to *β_1_* (i.e., the level of contact among <1 yr olds). Specifically, *β_2_* represents the contact among school-age children, the main age-group affected by measles during our study period (1951-2004). We used a prior of U[15, 70] for *β_2_* based on the average school class size in China (i.e., ~50 students per class [30]). According to a survey conducted in Guangzhou [31], the total number of contacts young adults encounter is comparable to that of children however less assortative (i.e., not as concentrated within the same age-group); we thus used a prior of U[10, 70] for *β_3_*, the within group mixing among 15-50 yr olds. Note that, although the priors were similar for *β_2_* and *β_3_*, additional school term time forcing (see Eqn 3 in the main text) applied to the former can substantially increase the level of mixing within school-age children to a level much higher than young adults. We used U[0.2, 1.2] for *β_4_* (within-group contact for >50 yr olds) and U[1, 5] for *β_5_* and *β_6_*, both of which represent child-parent mixing. The absolute transmission rates (i.e., the number of contacts times the probability of infection given contact) were computed using the eigenvalue approach (pp60-61 in ref [32]), which links *R_0_* and the ***β*** matrix*.*  That is, *R_0_* is the leading eigenvalue of the matrix ***nβ****D*, where *D* is the infectious period and ***n*** is a diagonal matrix with the fraction of population in each group (*n_i_*, *i*=1,…,4) on the diagonal.
6. Prior ranges for *b_1_*, the amplitude of school forcing. Three initial ranges were tested for all locations: U[0.4, 0.6], U[0.6, 0.8], and U[0.8, 1].
7. Prior ranges for *b.season*, the amplitude of seasonality. Five initial prior ranges were tested: U[0, 0.2], U[0.2, 0.3], U[0.3, 0.5], U[0.5, 0.7], and U[0.7, 1].
8. Latent period (*Z*) and infectious period (*D*). Based on previous studies [24,33], we used the following priors: U[7, 9] days for the latent period, and U[4, 6] days for the infectious period.

Taken together, in the first round of parameter selection, we tested 270 combinations (2 for *m_2_*, 3 for *r*, 3 for *R_0_*, 3 for *b_1_*, and 5 for *b.season*). The 'optimal' prior ranges after two rounds of selection are shown in Fig 5 and Fig S1.

**References:**

1. China.Com.Cn. (2009) Climate in Beijing (in Chinese). http://www.china.com.cn/aboutchina/zhuanti/09dfgl/2009-03/10/content_17415136.htm

2. National Bureau of Statistics of China. Census data. http://www.stats.gov.cn/english/statisticaldata/CensusData/

3. Wenfei Wang, Zhang W (2001) Population changes in Beijing since the economic reform (in Chinese). Population Research 25: 62-66.

4. Weather.Com.Cn. Introduction to Guangzhou city (in Chinese). http://www.weather.com.cn/html/cityintro/101280101.shtml

5. Guangzhou Bureau of Statistics. Statistics yearbook. http://210.72.4.52/gzStat1/chaxun/njsj.jsp

6. China Meteorological Administration. (2017) How was the north-versus-south region public heating provision desided? (in Chinese). http://www.cma.gov.cn/kppd/kppdrt/201712/t20171215_458231.html

7. Shandong Provincial Bureau of Statistics. Shandong statistics yearbook. http://www.stats-sd.gov.cn/col/col6279/index.html

8. Li J, Lu L, Pang XH, Sun MP, Ma R, et al. (2013) A 60-year review on the changing epidemiology of measles in capital Beijing, China, 1951-2011. Bmc Public Health 13.

9. Dai B, Chen Z, Liu Q, Wu T, Guo C, et al. (1991) Duration of immunity following immunization with live measles vaccine: 15 years of observation in zhejiang province, China. Bulletin of the World Health Organization 69: 415.

10. Yang Z, Xu J, Wang M, Di B, Tan H, et al. (2014) Measles epidemic from 1951 to 2012 and vaccine effectiveness in Guangzhou, southern China. Human vaccines & immunotherapeutics 10: 1091-1096.

11. Chan KW (2010) The household registration system and migrant labor in China: Notes on a debate. Popul Dev Rev 36: 357-364.

12. Information Center of Beijing Municipal Commission of Health and Family Planning. (2016) Changes in birthrate and death rate in Beijing during 1949-2015 (in Chinese). http://xxzx.bjchfp.gov.cn/tonjixinxi/weishengshujutiyao/jiankangzhibiao/201304/t20130425_60101.htm

13. Lin W, Xu R, Shao X, Yan X (2002) Epidemiological analysis and control strategies of measles in guangdong province (in Chinese). South China J Prev Med 28: 17-21.

14. Tian Z, Xiang H, Wu L (2003) Analysis of measels transmission charateristics in tianhe district, Guangzhou during 1985-2001 (in Chinese). South China J Prev Med 29: 27-28.

15. Jin S, Chen J (1997) Vaccination coverage in children in Guangzhou during 1986-1995 (in Chinese). Guangdong Journal of Health and Epidemic Prevention: 16-19.

16. Bufan Luo, Xiaoyun Li, Cai Y (2001) The changes of the ages on epidemiological characteristics of measles in Guangzhou and its trend analysis. Journal of Modern Clinical Medical Bioengineering 7: 345-347.

17. Yao H, Xu X, Xue D (2009) Analysis on the backgrounds and characteristics of floatiing population in Guangzhou. Tropical Geography 29: 350-355.

18. Xu A, Wang A, Song L, Liu G, Hao S, et al. (1995) Study on the epidemiological characteristics of measles in various stages and strategy for measles elimination in shandong province. Chinese Journal of Vaccines & Immunization 1: 22-25.

19. Li X, Kang D, Zhang Y, Wei G, Liu W, et al. (2012) Epidemic trend of measles in shandong province, China, 1963–2005. Public Health 126: 1017-1023.

20. Li S, Ma C, Hao L, Su Q, An Z, et al. (2017) Demographic transition and the dynamics of measles in six provinces in China: A modeling study. PLoS Med 14: e1002255.

21. Knoema. (2018) China - infant mortality rate. http://cn.knoema.com/atlas/中国/topics/人口统计资料/死亡率/婴儿死亡率

22. Beijing Bureau of Statistics (2012) The 2010 census in Beijing: China Statistics Press.

23. National Bureau of Statistics of China. (2002) Summary statistics for migrant populations in Beijing in 2001 (in Chinese). http://www.stats.gov.cn/tjsj/tjgb/qttjgb/dfqttjgb/200204/t20020404_30648.html

24. Keeling MJ, Grenfell BT (1997) Disease extinction and community size: Modeling the persistence of measles. Science 275: 65-67.

25. Finkenstadt BF, Grenfell BT (2000) Time series modelling of childhood diseases: A dynamical systems approach. J Roy Stat Soc C-App 49: 187-205.

26. Fine PE, Clarkson JA (1982) Measles in England and Wales--I: An analysis of factors underlying seasonal patterns. Int J Epidemiol 11: 5-14.

27. Keeling MJ, Grenfell BT (2002) Understanding the persistence of measles: Reconciling theory, simulation and observation. Proceedings Biological sciences / The Royal Society 269: 335-343.

28. Keeling MJ, Rohani P (2008) Temporally forced models. Modeling infectious diseases in humans and animals. 1st ed: Princeton University Press. pp. 155.

29. Anderson RM, May RM (1991) Infectious diseases of humans: Dynamics and control. Oxford: Oxford University Press.

30. Organization for Economic Cooperation and Development. Student-teach ratio and average class size. https://www.oecd-ilibrary.org/education/data/education-at-a-glance/student-teacher-ratio-and-average-class-size_334a3b64-en

31. Read JM, Lessler J, Riley S, Wang S, Tan LJ, et al. (2014) Social mixing patterns in rural and urban areas of southern China. Proceedings Biological sciences / The Royal Society 281: 20140268.

32. Keeling MJ, Rohani P (2008) Modeling infectious diseases in humans and animals: Princeton University Press.

33. Grenfell BT, Bolker BM (1998) Cities and villages: Infection hierarchies in a measles metapopulation. Ecology Letters 1: 63-70.

34. Beijing Bureau of Statistics. Resident population (1978-2014) (in Chinese). http://www.bjstats.gov.cn/rkjd/excel/czrk-7814.xls

Supplemental Tables

**S1 Table.** Summary of data type and source

| **Location** | **Data type** | **Time period** | **Time resolution** | **Data form** | **Source** |
| --- | --- | --- | --- | --- | --- |
| Beijing | Yearly incidence (entire population) | 1951-2011 | Annual | Figure | [8] |
| Beijing | Yearly incidence (1-14 yr olds) | 1978-2011 | Annual | Figure | [8] |
| Beijing | Incidence: monthly averages | 1985-1996; 1997-2004 | Multi-annual | Figure | [8] |
| Beijing | Vaccination: 1st dose | 1971-2011 | Annual | Figure | [8] |
| Beijing | Vaccination: 2nd dose | 1984-2011 | Annual | Figure | [8] |
| Beijing | Population: local, birthrate, death rate | 1951-2015 | Annual | Table | [12] |
| Beijing | Population: total, local, migrants, birthrate, death rate | 1978-2014 | Annual | Table | [34] |
| Beijing | Population: migrant age structure | 2010 | Annual | Text | [22] |
| Guangzhou | Yearly incidence (entire population) | 1951-2011 | Annual | Figure | [10] |
| Guangzhou | Incidence: monthly averages | 1965-2012 | Multi-annual | Figure | [10] |
| Guangzhou | Incidence: monthly averages in Guangdong province* | 1951-1966; 1967-1978; 1979-1986; 1987-2000 | Multi-annual | Table | [13] |
| Guangzhou | Incidence: monthly averages in Tianhe district** | 1985-2001 | Multi-annual | Table | [14] |
| Guangzhou | Vaccination: 1^st^ dose | 1986-1995 | Annual | Table | [15] |
| Guangzhou | Vaccination: 1^st^ dose | 1990, 1995, 1998-2016 | Annual | Table | [5] |
| Guangzhou | Population: local (registered), birth, death | 1949-2017 | Annual | Table | [5] |
| Guangzhou | Population: total (census data) | 1982, 1990, 2000, 2010 | Census | Table | [5] |
| Guangzhou | Population: migrant population size | 1989-2006 | Annual | Figure | [17] |
| Shandong | Yearly incidence (entire population) | 1951-1994 | Annual | Figure | [18] |
| Shandong | Yearly incidence (entire population) | 1963-2005 | Annual | Figure | [19] |
| Shandong | Yearly incidence (1-yr intervals for 0-10 yr olds and 5-yr intervals for 15+ yr olds) | 1985-2011 | Annual | Table | [20] |
| Shandong | Incidence: monthly averages | 1951-1966; 1967-1978; 1979-1989; 1990-1994 | Multi-annual | Table | [18] |
| Shandong | Vaccination: total vaccine doses used | 1967-1994 | Annual | Figure | [18] |
| Shandong | Population: local, total, birthrate, death rate | 1949-present | Annual | Table | [7] |
| China | Infant mortality rate | 1950-2015 | Annual | Figure | [21] |
| China | Population age structure | 1953, 1964, 1982, 1990, 2000 | Census | Table | [2] |

*Guangzhou is the capital city of Guangdong province; **Tianhe is a district within Guangzhou city.

**S2 Table.** Performance of the model-inference system using synthetic data.

| # truth | Correlation between model estimates and the synthetic truth | | | | Relative root-mean-square-error (RMSE) between model estimates and the synthetic truth | | | |
| --- | --- | --- | --- | --- | --- | --- | --- | --- |
|  | In-sample | Out-of-sample data (not used for fitting) | | | In-sample | Out-of-sample data (not used for fitting) | | |
|  | Yearly: All | Yearly: Group 2 | Weekly: All | Weekly: Group2 | Yearly: All | Yearly: Group 2 | Weekly: All | Weekly: Group2 |
| 1 | 1.00 | 1.00 | 0.89 | 0.88 | 0 | 0.06 | 1.40 | 1.52 |
| 2 | 1.00 | 1.00 | 0.90 | 0.89 | 0 | 0.04 | 1.24 | 1.30 |
| 3 | 1.00 | 1.00 | 0.91 | 0.91 | 0 | 0.05 | 1.21 | 1.25 |
| 4 | 1.00 | 1.00 | 0.91 | 0.90 | 0 | 0.06 | 1.32 | 1.41 |

Supplementary Figures

**S1 Fig.** Estimates of model parameters for the three locations during 1951-2004, not shown in Fig 5 in the main text: infectious period (A), latent period (B), and *β_2_* to *β_6_* (C-G). Solid lines show the mean posterior estimates and shaded areas show the 95% CIs; thick dashed lines show the prior ranges and thin dashed lines show the mean values of the priors.

**S2 Fig.** Synthetic truths. (A) Yearly incidence generated by the model using different combinations of *b_1_* and *b.season*. Solid lines show incidence for the entire population; these data were used as "observations" in the synthetic testing. Dashed lines show incidence for Group 2 (i.e. 1-14 yr olds). Monthly incidence aggregated for 1951-1966 (B), 1967-1978 (C), 1979-1984 (D), 1985-1996 (E), and 1997-2004 (F) were used for selection of the optimal priors.

**S3 Fig.** Results of synthetic testing of the model-inference system, using truth 1. (A) Model-fits to the observations (i.e. yearly incidence for the entire population). (B) Model estimates of incidence in the key age group (i.e. 1-14 yr olds); note these age-specific ‘truths’ were not used in model fitting. Model fits to monthly incidence aggregated for 1951-1966 (C), 1967-1978 (D), 1979-1984 (E), and 1985-1996 (F). These monthly aggregates were not directly used for model-fitting, but used to select the optimal parameter priors. Model estimates of *weekly* incidence for the entire population (G) and 1-14 yr olds (H), compared to the truth (not used for model-fitting). Estimates of key model parameters compared to the truth: *R_0_* (I), *m_2_* (J), reporting rate (K), *b_1_* (L), *b.season* (M) and *β_2_* to *β_6_* (N-R).

**S4 Fig.**  Same as S3 Fig but using truth 2.

**S5 Fig.** Same as S3 Fig but using truth 3.

**S6 Fig.** Same as S3 Fig but using truth 4.
