# Supplementary material for: Characteristics of measles epidemics in China (1951-2004) and implications for elimination: A case study of three key locations
Source: PLoS Comput Biol. 2019 Feb 4;15(2):e1006806. doi: 10.1371/journal.pcbi.1006806 (PMC6375639; doi:10.1371/journal.pcbi.1006806)
Supplement: S1 Table — (DOCX) [file pcbi.1006806.s002.docx]

**S1 Table.** Summary of data type and source

| **Location** | **Data type** | **Time period** | **Time resolution** | **Data form** | **Source** |
| --- | --- | --- | --- | --- | --- |
| Beijing | Yearly incidence (entire population) | 1951-2011 | Annual | Figure | [8] |
| Beijing | Yearly incidence (1-14 yr olds) | 1978-2011 | Annual | Figure | [8] |
| Beijing | Incidence: monthly averages | 1985-1996; 1997-2004 | Multi-annual | Figure | [8] |
| Beijing | Vaccination: 1st dose | 1971-2011 | Annual | Figure | [8] |
| Beijing | Vaccination: 2nd dose | 1984-2011 | Annual | Figure | [8] |
| Beijing | Population: local, birthrate, death rate | 1951-2015 | Annual | Table | [12] |
| Beijing | Population: total, local, migrants, birthrate, death rate | 1978-2014 | Annual | Table | [34] |
| Beijing | Population: migrant age structure | 2010 | Annual | Text | [22] |
| Guangzhou | Yearly incidence (entire population) | 1951-2011 | Annual | Figure | [10] |
| Guangzhou | Incidence: monthly averages | 1965-2012 | Multi-annual | Figure | [10] |
| Guangzhou | Incidence: monthly averages in Guangdong province* | 1951-1966; 1967-1978; 1979-1986; 1987-2000 | Multi-annual | Table | [13] |
| Guangzhou | Incidence: monthly averages in Tianhe district** | 1985-2001 | Multi-annual | Table | [14] |
| Guangzhou | Vaccination: 1^st^ dose | 1986-1995 | Annual | Table | [15] |
| Guangzhou | Vaccination: 1^st^ dose | 1990, 1995, 1998-2016 | Annual | Table | [5] |
| Guangzhou | Population: local (registered), birth, death | 1949-2017 | Annual | Table | [5] |
| Guangzhou | Population: total (census data) | 1982, 1990, 2000, 2010 | Census | Table | [5] |
| Guangzhou | Population: migrant population size | 1989-2006 | Annual | Figure | [17] |
| Shandong | Yearly incidence (entire population) | 1951-1994 | Annual | Figure | [18] |
| Shandong | Yearly incidence (entire population) | 1963-2005 | Annual | Figure | [19] |
| Shandong | Yearly incidence (1-yr intervals for 0-10 yr olds and 5-yr intervals for 15+ yr olds) | 1985-2011 | Annual | Table | [20] |
| Shandong | Incidence: monthly averages | 1951-1966; 1967-1978; 1979-1989; 1990-1994 | Multi-annual | Table | [18] |
| Shandong | Vaccination: total vaccine doses used | 1967-1994 | Annual | Figure | [18] |
| Shandong | Population: local, total, birthrate, death rate | 1949-present | Annual | Table | [7] |
| China | Infant mortality rate | 1950-2015 | Annual | Figure | [21] |
| China | Population age structure | 1953, 1964, 1982, 1990, 2000 | Census | Table | [2] |

*Guangzhou is the capital city of Guangdong province; **Tianhe is a district within Guangzhou city.
