# Supplementary material for: Characteristics of measles epidemics in China (1951-2004) and implications for elimination: A case study of three key locations
Source: PLoS Comput Biol. 2019 Feb 4;15(2):e1006806. doi: 10.1371/journal.pcbi.1006806 (PMC6375639; doi:10.1371/journal.pcbi.1006806)
Supplement: S2 Table — (DOCX) [file pcbi.1006806.s003.docx]

**S2 Table.** Performance of the model-inference system using synthetic data.

| # truth | Correlation between model estimates and the synthetic truth | | | | Relative root-mean-square-error (RMSE) between model estimates and the synthetic truth | | | |
| --- | --- | --- | --- | --- | --- | --- | --- | --- |
|  | In-sample | Out-of-sample data (not used for fitting) | | | In-sample | Out-of-sample data (not used for fitting) | | |
|  | Yearly: All | Yearly: Group 2 | Weekly: All | Weekly: Group2 | Yearly: All | Yearly: Group 2 | Weekly: All | Weekly: Group2 |
| 1 | 1.00 | 1.00 | 0.89 | 0.88 | 0 | 0.06 | 1.40 | 1.52 |
| 2 | 1.00 | 1.00 | 0.90 | 0.89 | 0 | 0.04 | 1.24 | 1.30 |
| 3 | 1.00 | 1.00 | 0.91 | 0.91 | 0 | 0.05 | 1.21 | 1.25 |
| 4 | 1.00 | 1.00 | 0.91 | 0.90 | 0 | 0.06 | 1.32 | 1.41 |
