# Supplementary figures and images for: Characteristics of measles epidemics in China (1951-2004) and implications for elimination: A case study of three key locations
Source: PLoS Comput Biol. 2019 Feb 4;15(2):e1006806. doi: 10.1371/journal.pcbi.1006806 (PMC6375639; doi:10.1371/journal.pcbi.1006806)

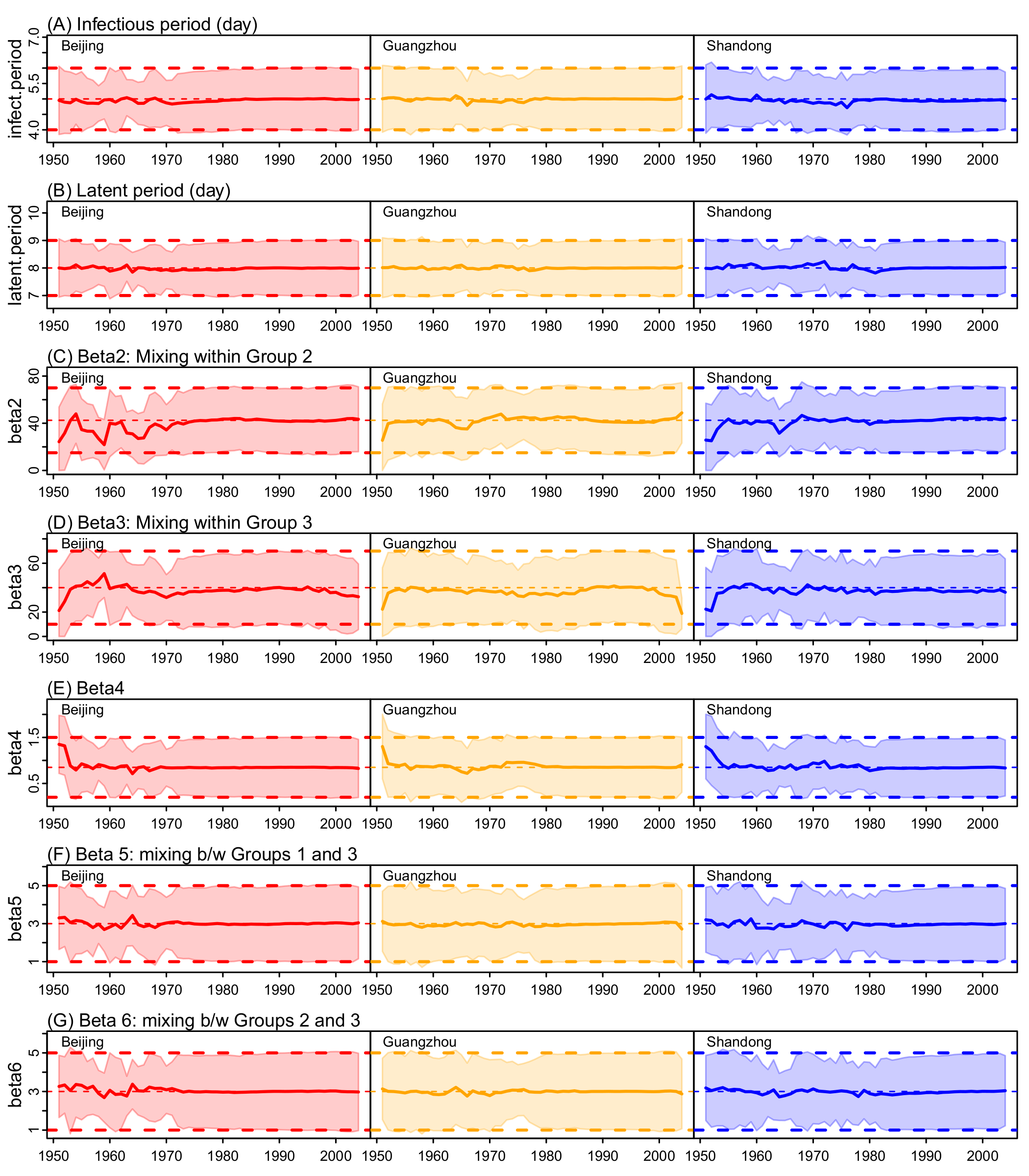

Supplement: S1 Fig — Estimates of model parameters for the three locations during 1951–2004, not shown in Fig 5 in the main text: infectious period (A), latent period (B), and β2 to β6 (C-G). Solid lines show the mean posterior estimates and shaded areas show the 95% CIs; thick dashed lines show the prior ranges and thin dashed lines show the mean values of the priors. (TIF) [file pcbi.1006806.s004.tif]

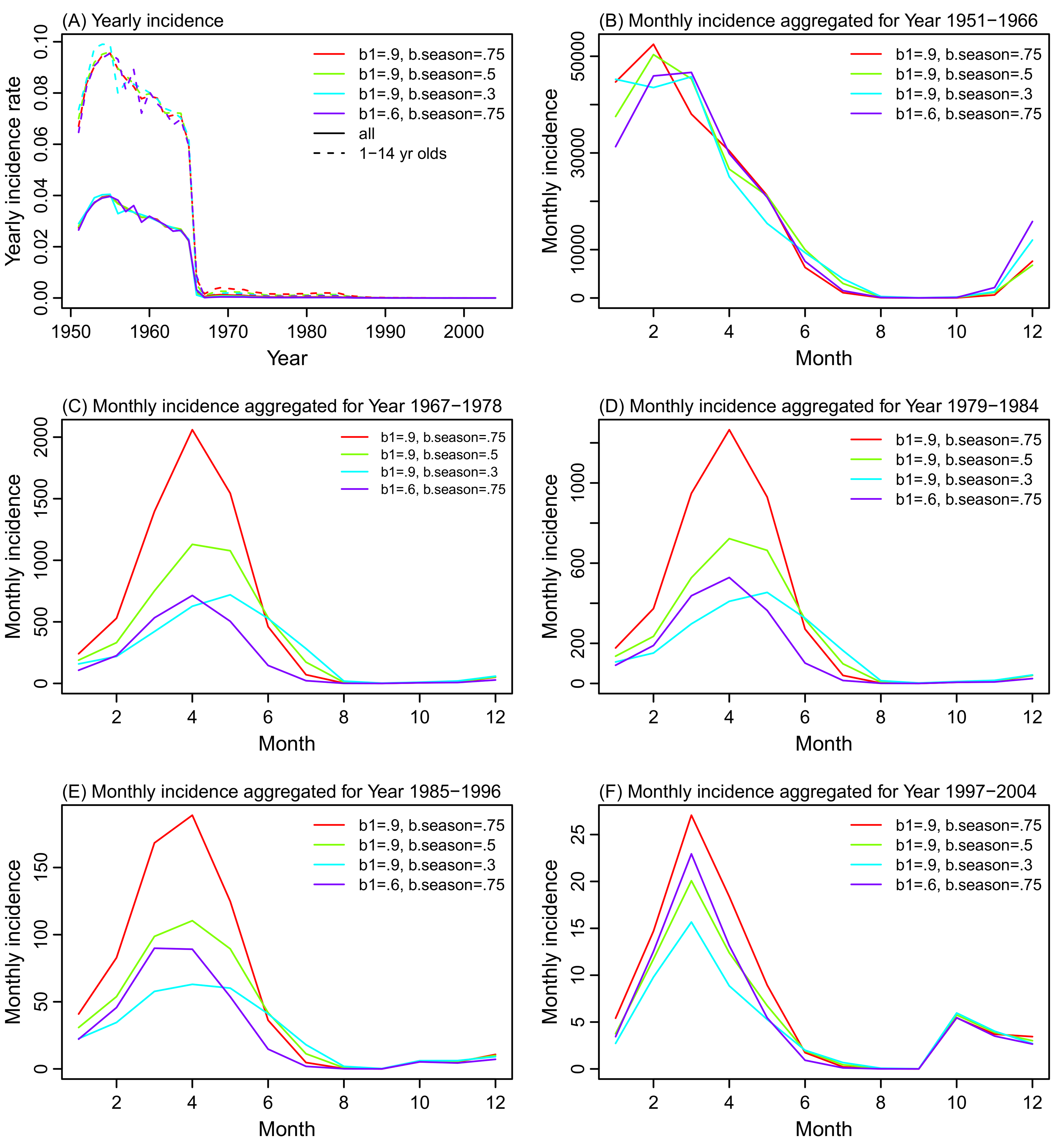

Supplement: S2 Fig — (A) Yearly incidence generated by the model using different combinations of b1 and b.season. Solid lines show incidence for the entire population; these data were used as "observations" in the synthetic testing. Dashed lines show incidence for Group 2 (i.e. 1–14 yr olds). Monthly incidence aggregated for 1951–1966 (B), 1967–1978 (C), 1979–1984 (D), 1985–1996 (E), and 1997–2004 (F) were used for selection of the optimal priors. (TIF) [file pcbi.1006806.s005.tif]

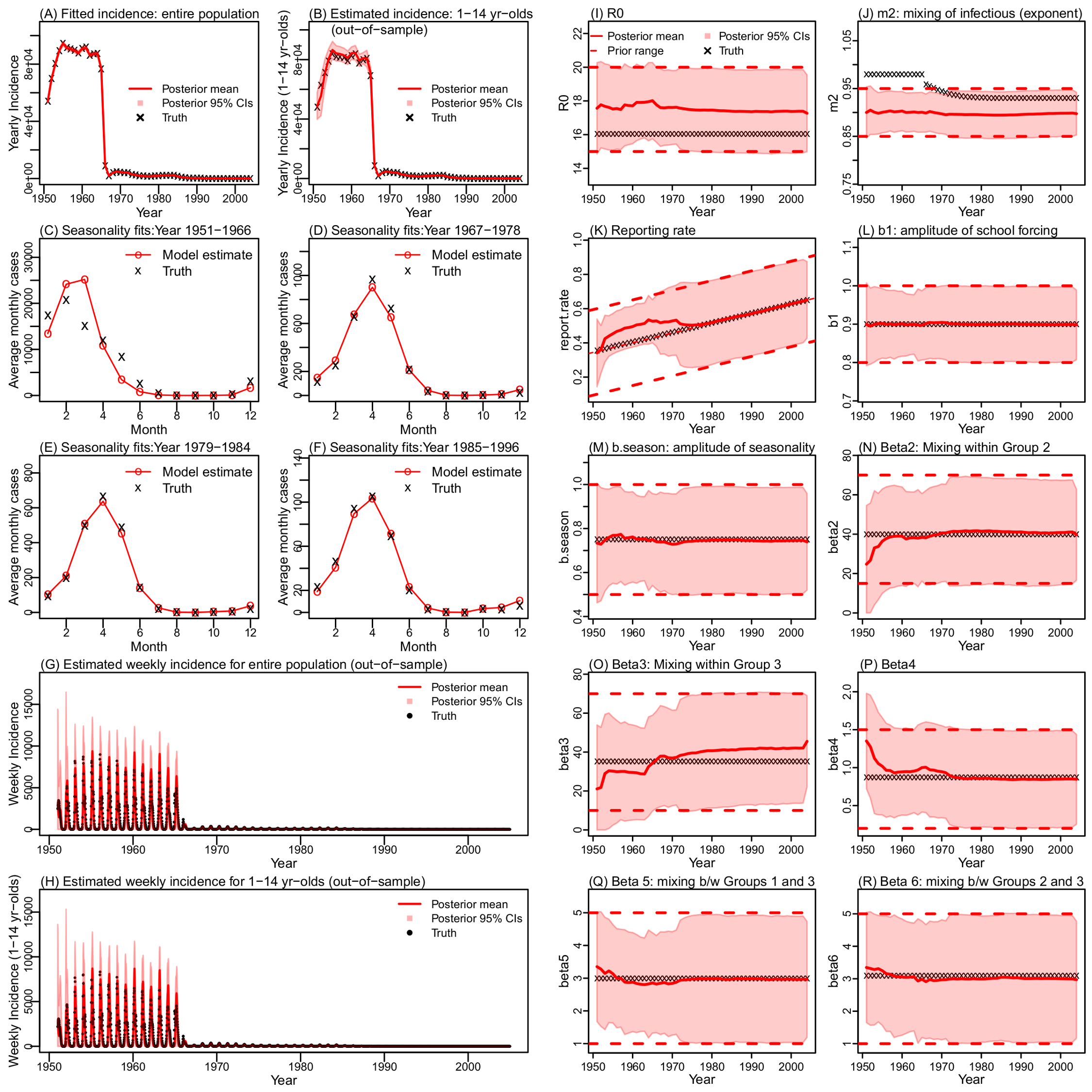

Supplement: S3 Fig — (A) Model-fits to the observations (i.e. yearly incidence for the entire population). (B) Model estimates of incidence in the key age group (i.e. 1–14 yr olds); note these age-specific ‘truths’ were not used in model fitting. Model fits to monthly incidence aggregated for 1951–1966 (C), 1967–1978 (D), 1979–1984 (E), and 1985–1996 (F). These monthly aggregates were not directly used for model-fitting, but used to select the optimal parameter priors. Model estimates of weekly incidence for the entire population (G) and 1–14 yr olds (H), compared to the truth (not used for model-fitting). Estimates of key model parameters compared to the truth: R0 (I), m2 (J), reporting rate (K), b1 (L), b.season (M) and β2 to β6 (N-R). (TIF) [file pcbi.1006806.s006.tif]

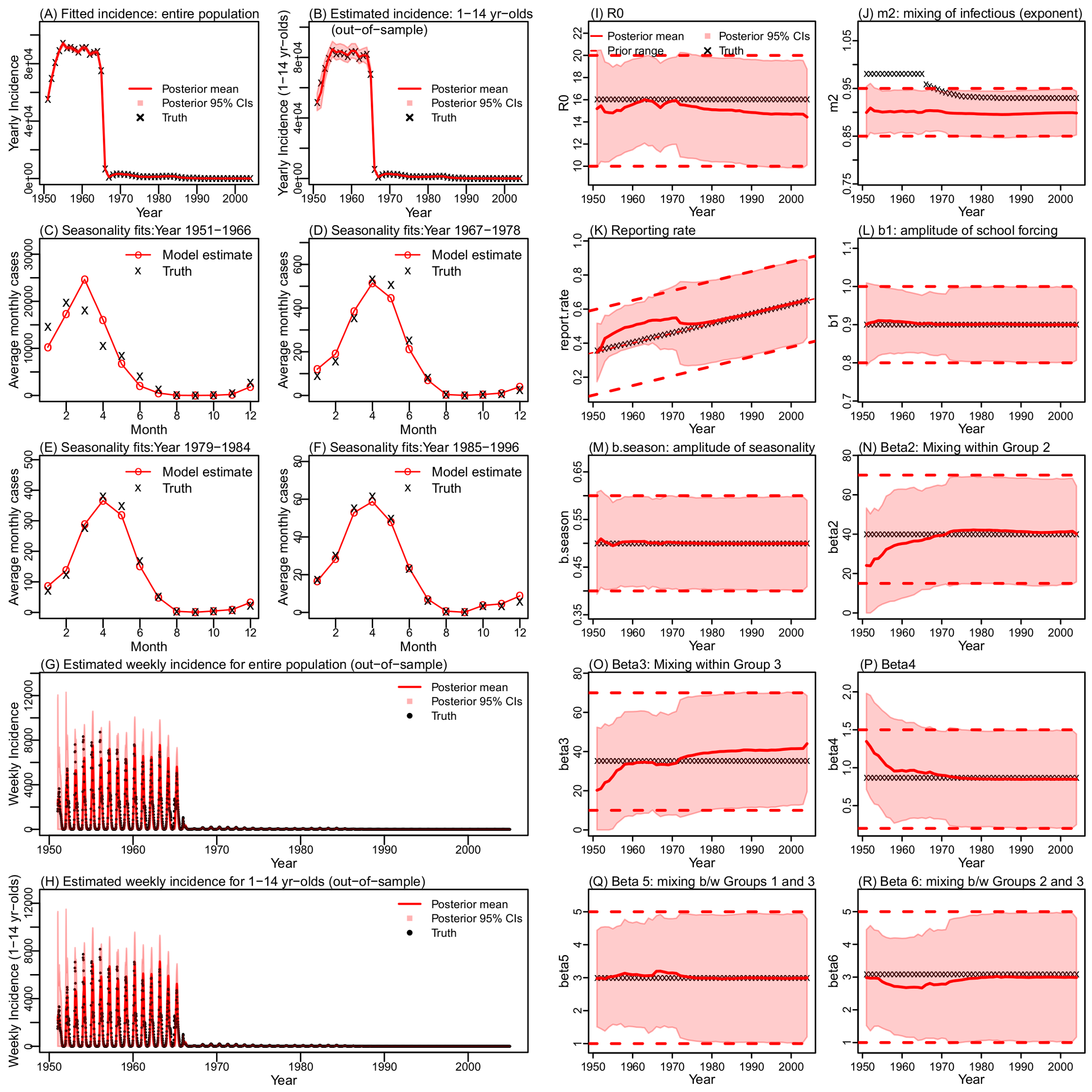

Supplement: S4 Fig — (A) Model-fits to the observations (i.e. yearly incidence for the entire population). (B) Model estimates of incidence in the key age group (i.e. 1–14 yr olds); note these age-specific ‘truths’ were not used in model fitting. Model fits to monthly incidence aggregated for 1951–1966 (C), 1967–1978 (D), 1979–1984 (E), and 1985–1996 (F). These monthly aggregates were not directly used for model-fitting, but used to select the optimal parameter priors. Model estimates of weekly incidence for the entire population (G) and 1–14 yr olds (H), compared to the truth (not used for model-fitting). Estimates of key model parameters compared to the truth: R0 (I), m2 (J), reporting rate (K), b1 (L), b.season (M) and β2 to β6 (N-R). (TIF) [file pcbi.1006806.s007.tif]

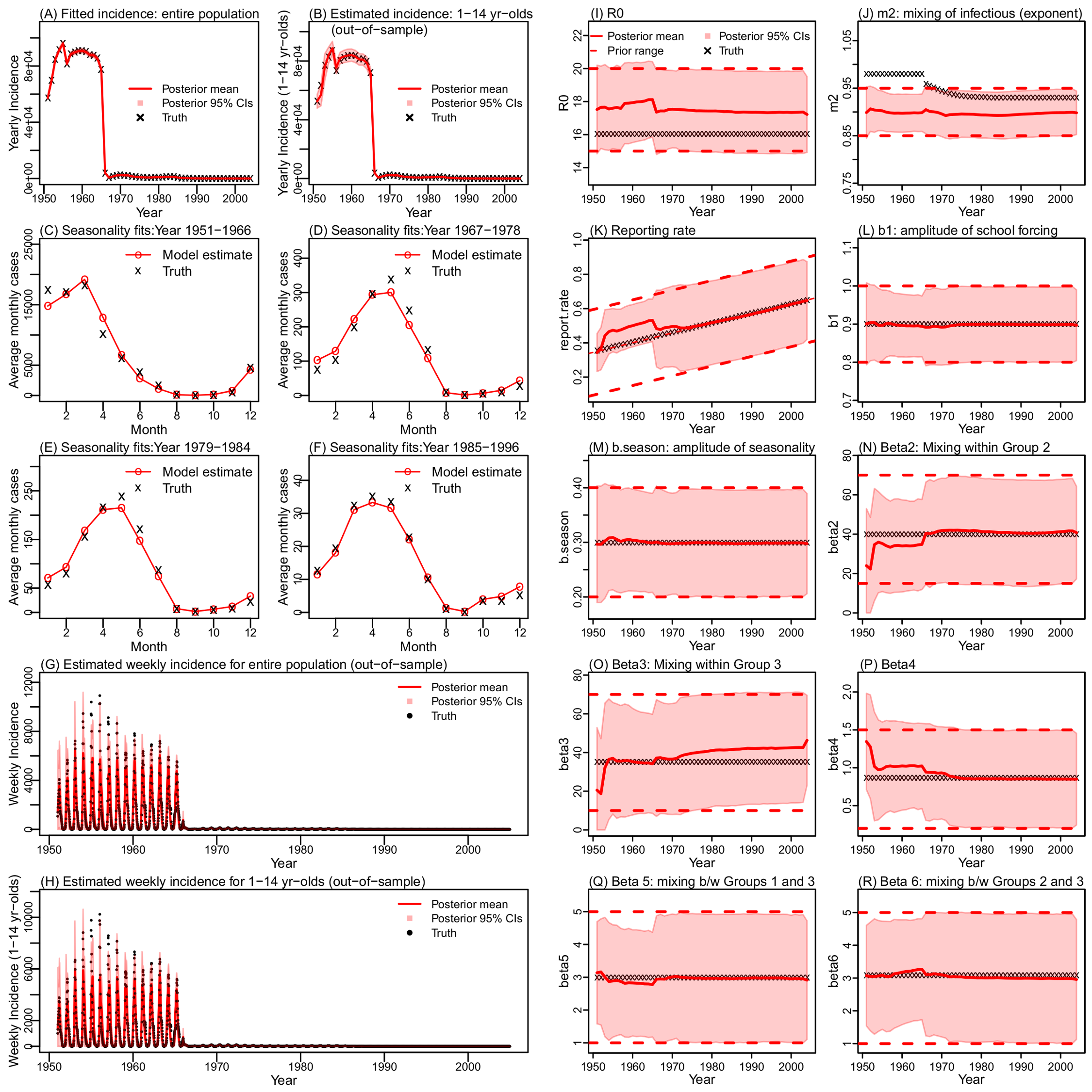

Supplement: S5 Fig — (A) Model-fits to the observations (i.e. yearly incidence for the entire population). (B) Model estimates of incidence in the key age group (i.e. 1–14 yr olds); note these age-specific ‘truths’ were not used in model fitting. Model fits to monthly incidence aggregated for 1951–1966 (C), 1967–1978 (D), 1979–1984 (E), and 1985–1996 (F). These monthly aggregates were not directly used for model-fitting, but used to select the optimal parameter priors. Model estimates of weekly incidence for the entire population (G) and 1–14 yr olds (H), compared to the truth (not used for model-fitting). Estimates of key model parameters compared to the truth: R0 (I), m2 (J), reporting rate (K), b1 (L), b.season (M) and β2 to β6 (N-R). (TIF) [file pcbi.1006806.s008.tif]

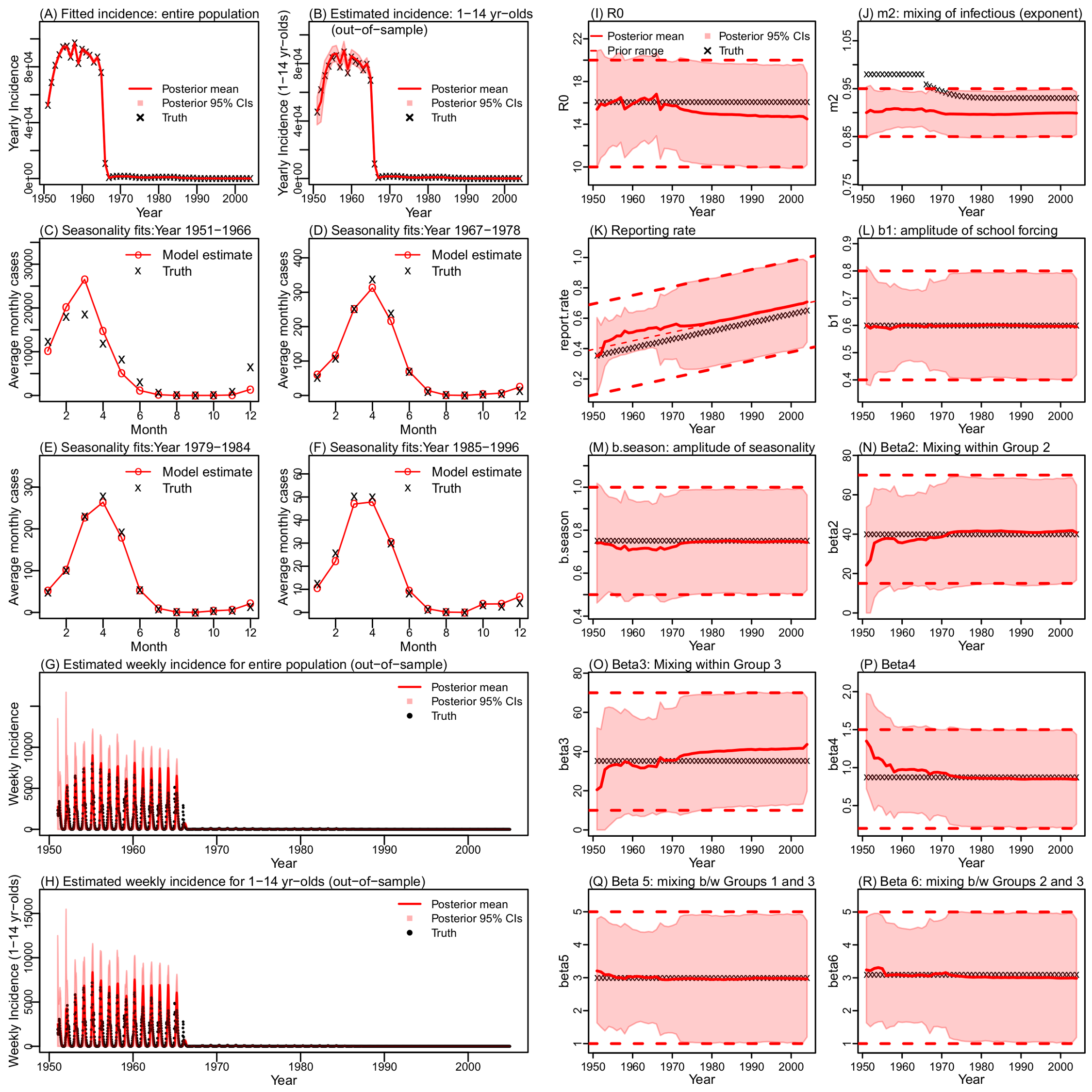

Supplement: S6 Fig — (A) Model-fits to the observations (i.e. yearly incidence for the entire population). (B) Model estimates of incidence in the key age group (i.e. 1–14 yr olds); note these age-specific ‘truths’ were not used in model fitting. Model fits to monthly incidence aggregated for 1951–1966 (C), 1967–1978 (D), 1979–1984 (E), and 1985–1996 (F). These monthly aggregates were not directly used for model-fitting, but used to select the optimal parameter priors. Model estimates of weekly incidence for the entire population (G) and 1–14 yr olds (H), compared to the truth (not used for model-fitting). Estimates of key model parameters compared to the truth: R0 (I), m2 (J), reporting rate (K), b1 (L), b.season (M) and β2 to β6 (N-R). (TIF) [file pcbi.1006806.s009.tif]
